# Outcomes of methylprednisolone as an adjunct to endovascular therapy in patients with diabetes and admission hyperglycemia

**DOI:** 10.3389/fendo.2026.1932813

**Published:** 2026-10-08

**Authors:** Lei Zhang, Beibei Zhang, Lingyu Zhang, Kaiyuan Feng, Linyu Li, Haoxuan Zhu, Xu Xu, Jinfu Ma, Sheng Zhou, Zhixi Wang, Jiayu Li, Qiuyi Yang, Yian Chen, Mingyang Chen, Yihui Yang, Yuhan Fan, Junjie Yuan, Jiaxing Song, Gaoming Li, Mingrui Zhou

**Affiliations:** 1 Department of Neurosurgery, The People's Hospital of Qiandongnan Miao and Dong Autonomous Prefecture, Kaili, Guizhou, China; 2 Department of Encephalopathy, Hubei Provincial Hospital of Traditional Chinese Medicine, Wuhan, Hubei, China; 3 Hubei Key Laboratory of theory and application research of liver and kidney in traditional Chinese medicine, Affiliated Hospital of Hubei University of Chinese Medicine, Wuhan, Hubei, China; 4 Hubei Shizhen Laboratory, Wuhan, Hubei, China; 5 Hubei Province Academy of Traditional Chinese Medicine, Wuhan, Hubei, China; 6 Department of Neurology, The Second Hospital & Clinical Medical School, Lanzhou University, Chongqing, China; 7 Department of Neurology, Xinqiao Hospital and The Second Affiliated Hospital, Army Medical University (Third Military Medical University), Chongqing, China; 8 Department of Neurology, The Second Affiliated Hospital of Chongqing Medical University, Chongqing, China; 9 Department of Neurology, The 908th Hospital of Chinese People’s Liberation Army Joint Logistic Support Force, Nanchang, Jiangxi, China; 10 Department of Neurology, Renhuai People’s Hospital, Zunyi, Guizhou, China; 11 The First Clinical Medical School, Hubei University of Chinese Medicine, Wuhan, Hubei, China

**Keywords:** acute ischemic stroke, admission hyperglycemia, clinical outcomes, diabetes, endovascular therapy, methylprednisolone

## Abstract

**Background:**

This study aimed to evaluate the effect of methylprednisolone as adjunctive treatment to endovascular therapy (EVT) in patients with and without diabetes, as well as in patients with and without admission hyperglycemia.

**Methods:**

This study was a secondary analysis of the MARVEL trial. The primary outcome was the ordinal modified Rankin Scale (mRS) score at 90 days. Secondary outcomes included mRS score of 0-3, 0-2, 0–1 at 90 days. Safety outcomes included mortality within 90 days, symptomatic intracranial hemorrhage (sICH) and any ICH within 48 hours.

**Results:**

Among 1,678 patients from the MARVEL trial, 321 had history of diabetes. In patients with diabetes, methylprednisolone was not associated with improved 90-day mRS [common odds radio (cOR), 1.07 (0.81-1.40), P = 0.640] but was associated with more any ICH within 48 hours [OR, 1.63 (1.17-2.26), P = 0.004]. In patients without diabetes, methylprednisolone was associated with better 90-day mRS [cOR, 1.16 (1.02-1.33), P = 0.026] and lower 90-day mortality [OR, 0.74 (0.62-0.88), P = 0.001]. A significant treatment-by-diabetes-history interaction was observed only for any ICH within 48 hours (P for interaction = 0.036). Among 1,470 patients stratified by admission hyperglycemia, methylprednisolone was not associated with improved 90-day mRS in either subgroup.

**Conclusion:**

The glycemia status-by-treatment interaction was not significant. A benefit trend was observed with adjunctive methylprednisolone in patients without diabetes. However, adjunctive use of methylprednisolone in patients with diabetes may be with a higher risk of any intracranial hemorrhage. Admission hyperglycemia status did not significantly modify the effectiveness or safety of methylprednisolone. These findings warrant validation in further studies.

## Introduction

As endovascular therapy (EVT) has become the standard treatment for acute ischemic stroke (AIS) with large vessel occlusion (LVO), approximately 50% of patients still experience poor functional outcomes, despite achieving recanalization rates exceeding 80% (1, 2). Therefore, how to further improve 90-day functional independence in patients following successful recanalization has emerged as a critical issue that urgently needs to be addressed (3). In recent years, neuroprotective and anti-inflammatory therapeutic strategies targeting reperfusion injury and post-ischemic inflammatory responses have become a frontier hotspot in stroke research, providing new directions for optimizing EVT efficacy (4, 5).

Methylprednisolone, a potent glucocorticoid with anti-inflammatory properties, has emerged as a promising adjunctive therapy for mitigating reperfusion injury (6, 7). The MARVEL (Methylprednisolone as Adjunct to Endovascular Thrombectomy for Large-Vessel Occlusion Stroke) randomized controlled trial demonstrated that intravenous methylprednisolone following EVT, compared with placebo, did not improve 90-day functional outcomes but significantly reduced mortality and symptomatic intracranial hemorrhage (sICH) (8). Nevertheless, methylprednisolone is known to exert considerable effects on glucose metabolism (9, 10). It can significantly elevate blood glucose levels by increasing hepatic gluconeogenesis, inhibiting peripheral glucose uptake, and inducing insulin resistance (9, 11). Moreover, admission hyperglycemia has been established as an independent risk factor for infarct expansion, hemorrhagic transformation, and poor functional outcomes in patients with AIS (12, 13). Building on this, diabetes mellitus, as an underlying condition characterized by chronic hyperglycemia and metabolic dysregulation, may not only exacerbate the aforementioned glycemic fluctuations induced by methylprednisolone, but also alter patients’ responses to glucocorticoid therapy through its distinct neurovascular pathological mechanisms (14, 15). However, the effect of methylprednisolone as adjunctive therapy to EVT in patients with with different glycemic states (acute or chronic hyperglycemia phase) remain unclear.

In this context, using the data from the MARVEL trial (8), this study aimed to evaluate the effect of methylprednisolone as adjunctive therapy to EVT in patients with different glycemic states.

## Methods

### Study design and participants

This study was a secondary analysis of the MARVEL trial, an investigator-initiated, randomized, double-blind, placebo-controlled clinical trial conducted at 82 stroke centers across China from February 9, 2022, to June 30, 2023, with follow-up for 3 months (8). The MARVEL trial aimed to assess the efficacy and adverse events of adjunctive intravenous low-dose methylprednisolone to endovascular thrombectomy for AIS secondary to LVO. Comprehensive information regarding the rationale, design, methods, and major findings of the MARVEL trial has been previously documented (8). This study followed the ethical principles of the Helsinki Declaration and was approved by the ethics committees of all participating centers. Written informed consent was provided by all patients or their legally authorized representative before randomization. This study was reported in accordance with the Consolidated Standards of Reporting Trials (CONSORT) guidelines for randomized trials.

Patients who met the following inclusion criteria were recruited to this study (1): age ≥ 18 years (2); The time from onset to randomization was within 24 hours (3); Anterior circulation ischemic stroke was preliminarily determined according to clinical symptoms or imaging examination (4); Baseline National Institutes of Health Stroke Scale (NIHSS) score ≥ 6 (5); Baseline Alberta Stroke Program Early CT Score (ASPECTS) ≥3 (6); Computed tomography angiography (CTA)/magnetic resonance angiography (MRA)/digital subtraction angiography (DSA) confirmed occlusion of the intracranial segment of the internal carotid artery or middle cerebral artery, and decision to undergo endovascular therapy. The exclusion criteria for this study included (1): pre-stroke mRS score ≥2 (2); Allergic to glucocorticoids (3); patients with severe or terminal illnesses; Detailed inclusion and exclusion criteria are provided in previously documented 8.

### Intervention and definition

All patients underwent EVT. Eligible patients were randomized to receive intravenous methylprednisolone at a dose of 2mg/kg/d (up to a maximum dose of 160mg) for 3 days vs placebo. We defined acute admission hyperglycemia as blood glucose levels ≥7.8 mmol/L. Chronic hyperglycemia is defined as a history of diabetes mellitus in the patient.

### Data collection

We collected the following clinical variables from the database: demographic characteristics, medical history, prestroke modified Rankin Scale (mRS) score, baseline glucose level, baseline stroke severity, extent of ischemic injury, stroke etiology, the site of occlusion, the use of intravenous thrombolysis, time metrics (last known to be well to puncture, last known to be well to randomization, last known to be well to recanalization, time from randomization to initial treatment) and blood pressure at hospital arrival. Baseline stroke severity was measured using the NIHSS score. The extent of ischemic injury was assessed with ASPECTS. Stroke etiology was categorized based on the Trial of ORG 10172 in Acute Stroke Treatment (TOAST) criteria.

### Outcomes

All participants underwent follow-up assessment at the 90-day timepoint, measured using the modified Rankin Scale (mRS) scores. Data collection was conducted either through in-person clinic visits or structured telephone interviews. To minimize assessment bias, mRS scoring was predominantly performed via centralized review of video or audio recordings by blinded outcome adjudicators. In circumstances where such recordings were not obtainable, face-to-face evaluation by blinded local investigators served as an alternative approach. The primary outcome was the distribution of the mRS score (ranging from 0 [no symptoms] to 6 [death]) at 90 days after randomization. Secondary outcomes included NIHSS score at 5 to 7 days or at early discharge; the following prespecified dichotomizations of the mRS score at 90 days: 0 to 3 vs 4 to 6, 0 to 2 vs 3 to 6 and 0 to 1 vs 2 to 6. Safety outcomes included all-cause mortality at 90 days, sICH within 48 hours, and any intracranial hemorrhage (ICH) within 48 hours. sICH was classified according to the modified Heidelberg Bleeding Classification.

### Statistical analysis

The Kolmogorov-Smirnov test was applied to assess the normality of continuous variables. As all continuous variables failed to meet the assumption of normal distribution, they were described using medians and interquartile ranges (IQR). Differences between groups in continuous variables were compared using the Mann-Whitney U test. Categorical variables were presented as frequencies and percentages, and differences between groups were analyzed using the chi-square test or Fisher’s exact test, as appropriate.

Logistic regression analysis were performed to evaluate clinical outcomes. For the distribution of mRS score, ordinal logistic regression was applied to estimate the common odds ratio (cOR) for a shift in mRS distribution toward better outcome. Binary logistic regression was performed for dichotomous outcomes, including mRS 0-3, mRS 0-2, mRS 0-1, mortality, sICH and any ICH. The NIHSS scores was analyzed by the linear regression with robust standard errors. In order to reduce the imbalance in confounders between treatment groups, the outcomes were adjusted using the inverse probability treatment weighting (IPTW) method (**Supplementary method 1**). Sample imputation was applied for the covariate to perform IPTW. (**Supplementary method 2**) (16) Missing data in the regression analysis were imputed using multiple imputation by chained equations. Ten imputed datasets were generated, and analysis were based on the combined results in accordance with Rubin’s rules (17). The variables included in the multiple imputation model were: admission hyperglycemia, group, age, baseline NIHSS score, pre-stroke mRS score, baseline ASPECTS, intravenous thrombolysis, onset-to-randomization time, occlusion site, 90-day mRS score, NIHSS score at 5–7 days, death, sICH and any ICH (18). The IPTW was calculated for patients adjusted for 7 covariates: age, baseline NIHSS score, pre-stroke mRS score, baseline ASPECTS, use of intravenous thrombolysis, time from stroke onset to randomization, and occlusion location, which are consistent with the covariates adjusted for in the multivariable logistic regression analysis. The estimates of odds ratios (OR), cOR, β coefficients, and 95% CIs were reported. The treatment effect of the history of diabetes on the primary outcome among patients with EVT plus methylprednisolone was assessed across different subgroups.

A two-tailed *P* value of less than 0.05 was considered statistically significant. All statistical analysis were performed using R software, version 4.4.2 (R Foundation for Statistical Computing).

## Results

### Baseline characteristics

A total of 1,680 patients were initially enrolled from the MARVEL trial. (**Supplementary Figure 1**) After excluding one patient with basilar artery occlusion and one patient with anterior cerebral artery occlusion, 1,678 patients were finally included in this study. Stratified by the history of diabetes, among the 1,678 total patients (321 with diabetes, 1,357 without), no significant baseline differences were observed between the methylprednisolone and placebo groups in the diabetes subgroup. However, in the non-diabetes group, the methylprednisolone group was slightly younger, had lower glucose levels at hospital arrival, and a lower prevalence of coronary heart disease compared with the placebo group. Further baseline characteristics were summarized in **Table 1**. Stratified by the admission hyperglycemia, among the 1,470 total patients (540 with admission hyperglycemia, 930 without admission hyperglycemia), no significant baseline differences were observed between the methylprednisolone and placebo groups. Further baseline characteristics were summarized in **Table 2**. Between-group comparisons after IPTW are detailed in **Supplementary Table 1** and **Supplementary Table 2**.

**Table 1:** Baseline characteristics of methylprednisolone versus placebo stratified by diabetes history before inverse probability of treatment weighting.

Characteristic	Diabetes (N = 321)				Without diabetes (N = 1357)			
	Methylprednisolone (N = 169)	Placebo (N = 152)	SMD	*P* value	Methylprednisolone (N = 668)	Placebo (N = 689)	SMD	*P* value
Age, median (IQR), y	68 (62-75)	69 (59-77)	0.002	0.953	68 (58-75)	70 (59-77)	0.128	0.016
Sex, man, No. (%)	88 (52.1)	84 (55.3)	0.064	0.567	391 (58.5)	389 (56.5)	0.042	0.440
Medical history^a^								
Hypertension	132 (78.1)	115 (75.7)	0.058	0.603	371 (55.5)	408 (59.2)	0.074	0.171
Atrial fibrillation	67 (39.6)	57 (37.5)	0.044	0.694	280 (41.9)	296 (43.0)	0.021	0.697
Hyperlipidemia	67 (39.6)	51 (33.6)	<0.001	0.258	198 (29.6)	194 (28.2)	<0.001	0.547
Coronary heart disease	44 (26.0)	32 (21.1)	0.127	0.294	103 (15.4)	137 (19.9)	0.033	0.031
Cerebral ischemia	29 (17.2)	26 (17.1)	0.118	0.990	107 (16.0)	91 (13.2)	0.117	0.143
Valvular heart disease	13 (7.7)	18 (11.8)	0.001	0.209	101 (15.1)	97 (14.1)	0.080	0.587
Intracranial hemorrhage	4 (2.4)	1 (0.7)	0.140	0.217	8 (1.2)	9 (1.3)	0.029	0.857
Transient ischemic attack	2 (1.2)	0 (0.0)	0.140	0.178	4 (0.6)	4 (0.6)	0.010	0.965
Smoking (current)	50 (29.6)	32 (21.1)	0.155	0.080	186 (27.8)	209 (30.3)	0.002	0.313
Prestroke modified Rankin Scale score 0-4^b^	10 (5.9)	9 (5.9)	0.197	0.999	26 (3.9)	15 (2.2)	0.055	0.065
Glucose level at hospital arrival, median (IQR), mmol/L	11.2 (8.4-14.4)	10.3 (8.2-13.8)	0.074	0.356	6.9 (6.1-8.1)	6.8 (5.8-7.8)	0.092	0.046
Baseline NIHSS score, median (IQR)^c^	19 (16-21)	18.5 (16-21)	0.051	0.553	19 (16-21)	19 (16-21)	0.010	0.767
Baseline ASPECTS, median (IQR)^d^	6 (4-8)	5 (4-7)	0.212	0.056	5 (4-7)	6 (4-7)	0.075	0.214
Stroke etiology^e^			0.133	0.706			0.118	0.197
Large artery atherosclerosis	83 (49.1)	80 (52.6)			219 (32.8)	254 (36.9)		
Cardioembolic	69 (40.8)	53 (34.9)			352 (52.7)	340 (49.3)		
Others	4 (2.4)	4 (2.6)			28 (4.2)	37 (5.4)		
Unknown	13 (7.7)	15 (9.9)			69 (10.3)	58 (8.4)		
Occlusion site^f^			0.105	0.644			0.131	0.056
ICA	53 (31.4)	52 (34.2)			233 (34.9)	241 (35.0)		
M1	89 (52.7)	81 (53.3)			363 (54.3)	345 (50.1)		
M2	27 (16.0)	19 (12.5)			72 (10.8)	103 (14.9)		
Intravenous thrombolysis	58 (34.3)	55 (36.2)	0.039	0.727	236 (35.3)	278 (40.3)	0.104	0.057
Time from last known well, median (IQR), min								
To puncture	344 (225-560)	380 (242-621)	0.045	0.375	349 (235-625)	355 (236-590)	0.022	0.786
To randomization	361 (237-579)	383 (247.5-611)	0.013	0.322	354.5 (239.8-634.2)	362 (238-581)	0.033	0.555
To recanalization	444 (303-648)	457 (316-706)	0.033	0.467	431 (318-710)	442 (311-688)	0.015	0.827
Time from randomization to initial treatment, median (IQR), h	8 (6-13)	8 (6-14)	0.108	0.606	8 (6-14)	8 (6-13)	0.085	0.461
Blood pressure at hospital arrival, median (IQR), mm Hg								
Systolic	147 (130-165)	150.5 (133-162)	0.018	0.641	140 (126-158)	143 (126-160)	0.029	0.370
Diastolic	83 (75-95)	86 (76-95)	0.095	0.309	82.5 (74-92)	83 (74-94)	0.051	0.317

a Comorbidities based on family or patient report.

b Scores on the mRS of functional disability range from 0 (no symptoms) to 6 (death).

c Scores on the NIHSS range from 0 to 42, with higher scores indicating more severe neurologic deficit.

d The ASPECTS is an imaging measure of the extent of ischemic stroke. Scores range from 0 to 10, with higher scores indicating a smaller infarct core. Listed are values for the core laboratory assessment. Data were missing for 6 in patients without diabetes.

e Stroke etiology was categorized according to the Trial of ORG 10172 in Acute Stroke Treatment (TOAST) classification.

f One patient had a basilar artery occlusion and 1 patient had an anterior cerebral artery occlusion in the methylprednisolone group.

ASPECTS, Alberta Stroke Program Early CT Score; ICA, Intracranial internal carotid artery; IQR, Interquartile range;

M1, M1 middle cerebral artery segment; M2, M2 middle cerebral artery segment; mRS, modified Rankin Scale; NIHSS, National Institutes of Health Stroke Scale; SMD, Standardized mean differences; TOAST, Trial of ORG 10172 in Acute Stroke Treatment.

**Table 2:** Baseline characteristics of methylprednisolone versus placebo stratified by admission hyperglycemia before inverse probability of treatment weighting.

Characteristic	Admission hyperglycemia (N = 540)				No admission hyperglycemia (N = 930)			
	Methylprednisolone (N = 283)	Placebo (N = 257)	SMD	*P* value	Methylprednisolone (N = 451)	Placebo (N = 479)	SMD	*P* value
Age, median (IQR), y	70 (62-76)	71 (60-77)	0.068	0.309	68 (58-75)	69 (59-77)	0.123	0.060
Sex, man, No. (%)	141 (49.8)	124 (48.2)	0.031	0.715	275 (61.0)	296 (61.8)	0.017	0.797
Medical history^a^								
Hypertension	192 (67.8)	189 (73.5)	0.125	0.147	255 (56.5)	283 (59.1)	0.051	0.433
Atrial fibrillation	121 (42.8)	121 (47.1)	0.087	0.313	184 (40.8)	190 (39.7)	0.023	0.725
Hyperlipidemia	97 (34.3)	79 (30.7)	0.035	0.381	144 (31.9)	141 (29.4)	0.006	0.410
Diabetes	114 (40.3)	108 (42.0)	0.076	0.681	28 (6.2)	29 (6.1)	0.054	0.922
Coronary heart disease	64 (22.6)	62 (24.1)	0.036	0.679	66 (14.6)	85 (17.7)	0.085	0.199
Cerebral ischemia	41 (14.5)	39 (15.2)	0.019	0.822	79 (17.5)	62 (12.9)	0.128	0.052
Valvular heart disease	33 (11.7)	28 (10.9)	0.024	0.779	62 (13.7)	73 (15.2)	0.042	0.518
Intracranial hemorrhage	5 (1.8)	4 (1.6)	0.016	0.849	7 (1.6)	4 (0.8)	0.066	0.312
Transient ischemic attack	2 (0.7)	0 (0.0)	0.119	0.177	3 (0.7)	4 (0.8)	0.020	0.765
Smoking (current)	65 (23.0)	56 (21.8)	0.028	0.743	138 (30.6)	147 (30.7)	0.002	0.976
Prestroke modified Rankin Scale score 0-4^b^	13 (4.6)	8 (3.1)	0.077	0.374	17 (3.8)	13 (2.7)	0.060	0.363
Glucose level at hospital arrival, median (IQR), mmol/L	9.5 (8.6-12.0)	9.8 (8.5-12.1)	0.009	0.872	6.4 (5.8-7.0)	6.3 (5.6-7.0)	0.104	0.178
Baseline NIHSS score, median (IQR)^c^	19 (16-21)	20 (17-21)	0.119	0.168	19 (17-21)	19 (16-21)	0.012	0.677
Baseline ASPECTS, median (IQR)^d^	5 (4-7)	5 (4-7)	0.031	0.676	6 (4-7)	6 (4-7)	0.016	0.894
Stroke etiology^e^			0.084	0.816			0.131	0.266
Large artery atherosclerosis	108 (38.2)	108 (42.0)			154 (34.1)	187 (39.0)		
Cardioembolic	140 (49.5)	119 (46.3)			229 (50.8)	226 (47.2)		
Others	13 (4.6)	10 (3.9)			17 (3.8)	23 (4.8)		
Unknown	22 (7.8)	20 (7.8)			51 (11.3)	43 (9.0)		
Occlusion site^f^			0.161	0.174			0.084	0.441
ICA	103 (36.4)	86 (33.5)			151 (33.5)	168 (35.1)		
M1	152 (53.7)	132 (51.4)			244 (54.1)	241 (50.3)		
M2	28 (9.9)	39 (15.2)			56 (12.4)	70 (14.6)		
Intravenous thrombolysis	106 (37.5)	100 (38.9)	0.030	0.728	151 (33.5)	188 (39.2)	0.120	0.068
Time from last known well, median (IQR), min								
To puncture	320 (225-544)	350 (240-561)	0.024	0.349	375 (250-670)	376 (238-616)	0.049	0.332
To randomization	329 (228-557)	353 (238-582)	0.028	0.698	377 (254-710)	379 (253-600)	0.048	0.385
To recanalization	415 (306-627)	427 (306-656)	0.052	0.820	45 (322-763)	466 (321-730)	0.028	0.560
Time from randomization to initial treatment, median (IQR), h	8 (6-13)	8 (6-14)	0.076	0.192	8 (6-14)	8 (6-14)	0.117	0.127
Blood pressure at hospital arrival, median (IQR), mm Hg								
Systolic	148 (132-165)	149 (132-163)	0.009	0.815	140 (125-157)	143 (126-160)	0.046	0.271
Diastolic	82 (75-95)	85 (75-96)	0.117	0.221	84 (73-92)	84 (75-94)	0.043	0.399

a Comorbidities based on family or patient report.

b Scores on the mRS of functional disability range from 0 (no symptoms) to 6 (death).

c Scores on the NIHSS range from 0 to 42, with higher scores indicating more severe neurologic deficit.

d The ASPECTS is an imaging measure of the extent of ischemic stroke. Scores range from 0 to 10, with higher scores indicating a smaller infarct core. Listed are values for the core laboratory assessment. Data were missing for 2 patients with admission hyperglycemia and 4 patients with no admission hyperglycemia.

e Stroke etiology was categorized according to the Trial of ORG 10172 in Acute Stroke Treatment (TOAST) classification.

f One patient had a basilar artery occlusion and 1 patient had an anterior cerebral artery occlusion in the methylprednisolone group.

ASPECTS, Alberta Stroke Program Early CT Score; ICA, Intracranial internal carotid artery; IQR, Interquartile range;

M1, M1 middle cerebral artery segment; M2, M2 middle cerebral artery segment; mRS, modified Rankin Scale; NIHSS, National Institutes of Health Stroke Scale; SMD, Standardized mean differences; TOAST, Trial of ORG 10172 in Acute Stroke Treatment.

### Clinical outcomes

#### Stratified by diabetes history(methylprednisolone versus placebo)

Among patients with diabetes, methylprednisolone was not associated with significant improvement in the 90-day mRS score compared with placebo (IPTW-adjusted OR, 1.07 [95% CI, 0.81–1.40]; *P* = 0.640), nor in any secondary functional outcome (all *P* > 0.05). Safety outcomes did not differ significantly for 90-day mortality (IPTW-adjusted OR, 0.89 [95% CI, 0.62–1.27]; *P* = 0.526) or sICH within 48 hours (IPTW-adjusted OR, 0.96 [95% CI, 0.56–1.66]; *P* = 0.880), although any ICH within 48 hours was significantly more frequent with methylprednisolone (IPTW-adjusted OR, 1.63 [95% CI, 1.17–2.26]; *P* = 0.004) (**Table 3****).**

**Table 3:** Outcomes according to the treatment in patients with and without history of diabetes.

Outcomes		Methylprednisolone	Placebo	Unadjusted Effect value (95% CI)	P value	Adjusted Effect value (95% CI) ^a^	*P* value	IPTW adjusted value (95% CI) ^a^	*P* value	*P* for interaction
Primary outcomes										
mRS score at 90 days^b^	Diabetes	3 (2-5)/168	4 (2-6)/152	1.07 (0.73 - 1.58)	0.726	1.01 (0.68-1.50)	0.964	1.07 (0.81-1.40)	0.640	0.296
	Without Diabetes	3 (1-5)/665	3 (1-6)/686	1.18 (0.98 - 1.43)	0.081	1.20 (0.99-1.46)	0.061	1.16 (1.02-1.33)	0.026	
Secondary outcomes										
mRS 0 to 4 at 90 days	Diabetes	114/168 (67.9)	100/152 (65.8)	1.10 (0.69 - 1.75)	0.695	1.08 (0.65-1.79)	0.772	1.10 (0.79-1.53)	0.560	0.331
	Without Diabetes	482/665 (72.5)	455/686 (66.3)	1.34 (1.06 - 1.69)	0.014	1.37 (1.06-1.77)	0.017	1.31 (1.11-1.54)	0.001	
mRS 0 to 3 at 90 days	Diabetes	89/168 (53.0)	75/152 (49.3)	1.16 (0.75 - 1.79)	0.516	1.14 (0.70-1.85)	0.599	1.15 (0.84-1.56)	0.386	0.632
	Without Diabetes	406/665 (61.1)	384/686 (56.0)	1.23 (0.99 - 1.53)	0.058	1.24 (0.98-1.59)	0.079	1.20 (1.03-1.40)	0.019	
mRS 0 to 2 at 90 days	Diabetes	67/168 (39.9)	54/152 (35.5)	1.20 (0.76 - 1.89)	0.423	1.20 (0.73-1.95)	0.475	1.17 (0.85-1.61)	0.339	0.989
	Without Diabetes	300/665 (45.1)	289/686 (42.1)	1.13 (0.91 - 1.40)	0.269	1.14 (0.90-1.44)	0.285	1.11 (0.95-1.29)	0.173	
mRS 0 to 1 at 90 days	Diabetes	37/168 (22.0)	37/152 (24.3)	0.88 (0.52 - 1.48)	0.623	0.88 (0.51-1.53)	0.656	0.88 (0.61-1.27)	0.485	0.377
	Without Diabetes	182/665 (27.4)	183/686 (26.7)	1.04 (0.81 - 1.32)	0.775	1.05 (0.81-1.36)	0.720	1.03 (0.87-1.22)	0.730	
NIHSS score at 5–7 days^c^	Diabetes	13 (6-29)/169	12 (5-28)/152	0.40 (-2.56 - 3.36)	0.788	0.90 (-1.85-3.66)	0.518	0.87 (-2.08-3.83)	0.562	0.123
	Without Diabetes	10 (3-22)/667	12 (4-28)/687	-1.42 (-2.89 - 0.05)	0.058	-1.45 (-2.78--0.12)	0.033	-1.44 (-2.91-0.03)	0.054	
	Diabetes	12863/25688 (50.1)/152	11959/25688 (46.6)/169	0.93 (0.72 - 1.21)	0.586			WR:0.90 (0.69 - 1.17)	0.425	0.144
	Without Diabetes	207266/458229 (45.2)/687	232496/458229 (50.7)/667	1.12 (0.99 - 1.28)	0.079			WR:1.12 (0.99 - 1.28)	0.079	
Safety outcomes										
Mortality within 90 days^d^	Diabetes	41/168 (24.4)	40/152 (26.3)	0.90 (0.55 - 1.50)	0.695	0.91 (0.53-1.56)	0.728	0.89 (0.62-1.27)	0.526	0.327
	Without Diabetes	152/665 (22.9)	199/686 (29.0)	0.73 (0.57 - 0.93)	0.010	0.71 (0.54-0.92)	0.011	0.74 (0.62-0.88)	0.001	
sICH within 48 hours	Diabetes	15/168 (8.9)	14/150 (9.3)	0.95 (0.44 - 2.04)	0.900	0.97 (0.45-2.12)	0.947	0.96 (0.56-1.66)	0.880	0.339
	Without Diabetes	56/653 (8.6)	83/680 (12.2)	0.67 (0.47 - 0.96)	0.031	0.66 (0.46-0.95)	0.024	0.66 (0.51-0.85)	0.001	
Any ICH within 48 hours	Diabetes	67/168 (39.9)	45/150 (30.0)	1.55 (0.97 - 2.47)	0.066	1.64 (1.02-2.64)	0.043	1.63 (1.17-2.26)	0.004	0.036
	Without Diabetes	247/653 (37.8)	263/680 (38.7)	0.96 (0.77 - 1.20)	0.749	0.95 (0.76-1.19)	0.650	0.95 (0.81-1.11)	0.482	

a Values adjusted for age, baseline NIHSS score, prestroke mRS score, baseline ASPECTS, use of intravenous thrombolysis, time from onset to randomization, and occlusion location.

b Common odds ratio; The common odds ratio was estimated from an ordinal logistic regression model. The mRS of functional disability ranges from 0 (no symptoms) to 6 (death). Data were not available for 1 patient in the diabetes group and 6 patients in the no diabetes group.

c The β values were estimated from a multivariable linear regression model. The win proportion was calculated as the number of wins for the methylprednisolone group over the control group in NIHSS scores among all possible pairs of NIHSS scores (taking 1 patient from the methylprednisolone group and 1 patient from the control group), divided by the total number of pairs. The results were adjusted using the inverse probability of treatment weighting method. NIHSS scores range from 0 to 42; higher scores indicate more severe neurological deficits.

d sICH was defined according to the Heidelberg bleeding classification (an increase in the NIHSS score of 4 points or an increase in the score for an NIHSS subcategory of 2 points with any intracranial hemorrhage on imaging results).

CI, confidence interval; ICH, intracranial hemorrhage; IPTW, inverse probability of treatment weighting; IQR, interquartile range; mRS, modified Rankin Scale; NIHSS, National Institutes of Health Stroke Scale; OR, odds ratio; sICH, symptomatic intracranial hemorrhage.

Among patients without diabetes, methylprednisolone was associated with significantly better 90-day mRS scores (IPTW-adjusted OR, 1.16 [95% CI, 1.02–1.33]; *P* = 0.026) and higher rates of mRS 0–4 (IPTW-adjusted OR, 1.31 [95% CI, 1.11–1.54]; *P* = 0.001) and mRS 0–3 (IPTW-adjusted OR, 1.20 [95% CI, 1.03–1.40]; *P* = 0.019), as well as significantly lower 90-day mortality (IPTW-adjusted OR, 0.74 [95% CI, 0.62–0.88]; *P* = 0.001) and sICH within 48 hours (IPTW-adjusted OR, 0.66 [95% CI, 0.51–0.85]; *P* = 0.001). A significant treatment-by-diabetes-history interaction was observed only for any ICH within 48 hours (*P* for interaction = 0.036). No significant interactions were found for other outcomes (**Table 3**). The distribution of the modified Rankin Scale scores at 90 days by the history of diabetes, stratified by treatment arm was shown in **Figure 1A**. 

**Figure 1:**
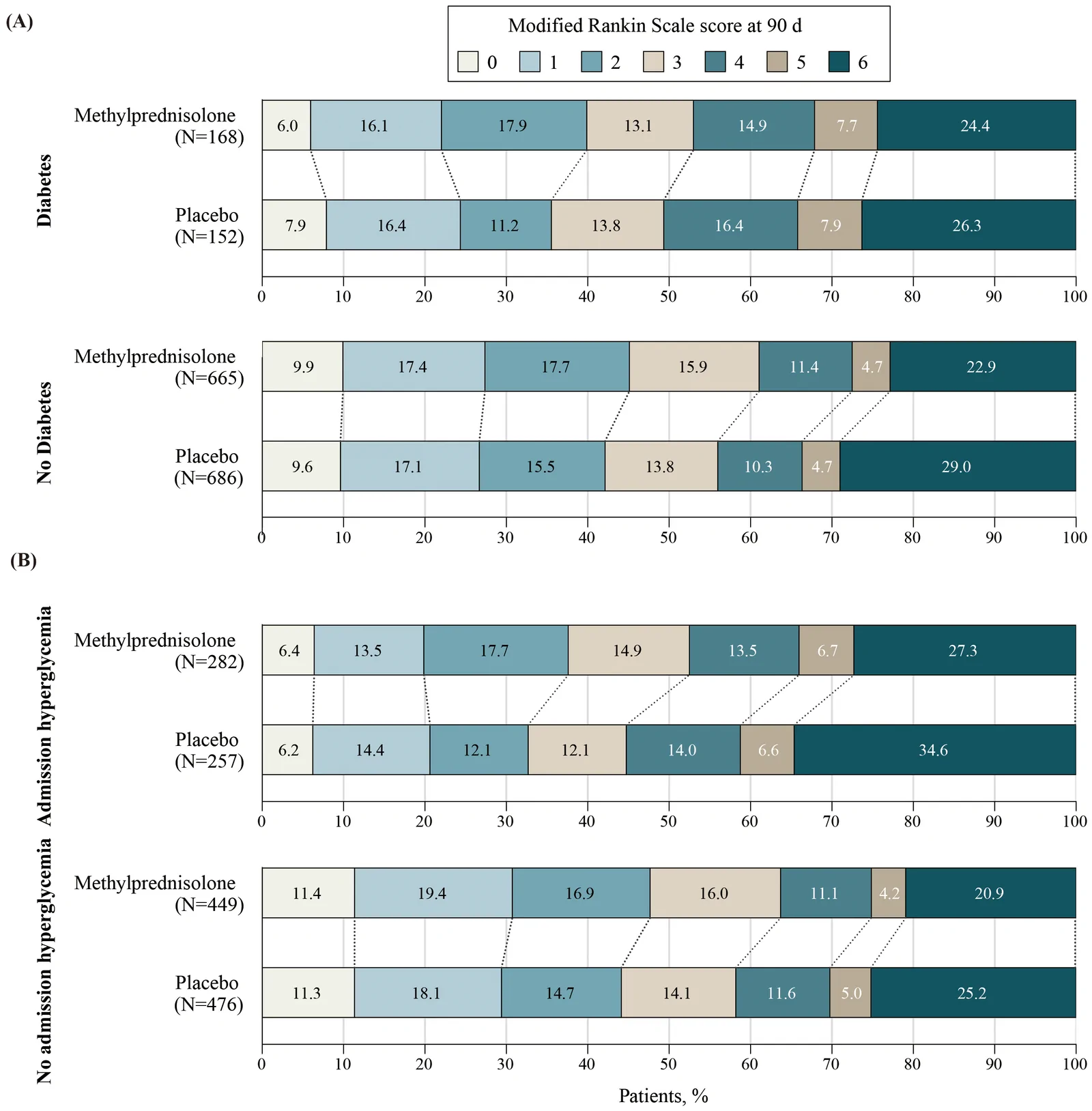
Distribution of the modified Rankin Scale score at 90 days. **(A)** The distribution of the modified Rankin Scale scores at 90 days by the history of diabetes, stratified by treatment arm. **(B)** The distribution of the modified Rankin Scale scores at 90 days by admission hyperglycemia, stratified by treatment arm. Scores on the modified Rankin Scale range from 0 to 6, with 0 indicating no symptoms; 1, symptoms without clinical disability; 2, slight disability; 3, moderate disability; 4, moderately severe disability; 5, severe disability; and 6, death.

#### Stratified by admission hyperglycemia(Methylprednisolone Versus Placebo)

Among patients with admission hyperglycemia, methylprednisolone was not associated with significant improvement in the 90-day mRS score compared with placebo (IPTW-adjusted OR, 1.17 [95% CI, 0.95–1.45]; *P* = 0.139), nor in any secondary functional outcome (all *P* > 0.05). Safety outcomes also did not differ significantly for 90-day mortality, sICH within 48 hours or any ICH within 48 hours (all *P* > 0.05). Among patients without admission hyperglycemia, methylprednisolone was not significantly associated with the 90-day mRS score (IPTW-adjusted OR, 1.13 [95% CI, 0.96–1.33]; *P* = 0.138), but was associated with significantly higher rates of mRS 0–4 (IPTW-adjusted OR, 1.24 [95% CI, 1.01–1.52]; *P* = 0.040) and mRS 0–3 (IPTW-adjusted OR, 1.21 [95% CI, 1.01–1.46]; *P* = 0.042). No significant differences were observed for safety outcomes, including 90-day mortality, sICH within 48 hours, and any ICH within 48 hours in patients with or without admission hyperglycemia. No significant treatment-by-admission-hyperglycemia interactions were observed for any outcome (all *P* for interaction > 0.05) (**Table 4**). The distribution of the modified Rankin Scale scores at 90 days by admission hyperglycemia, stratified by treatment arm was shown in **Figure 1B**.

**Table 4:** Outcomes according to the treatment in patients with and without admission hyperglycemia.

Outcomes		Methylprednisolone	Placebo	Unadjusted Effect value (95% CI)	P value	Adjusted Effect value (95% CI) ^a^	*P* value	IPTW adjusted value (95% CI) ^a^	*P* value	*P* for interaction
Primary outcomes										
mRS score at 90 days^b^	Admission hyperglycemia	3 (2-6)/282	4 (2-6)/257	1.27 (0.94 - 1.71)	0.122	1.18 (0.87-1.61)	0.291	1.17 (0.95-1.45)	0.139	0.828
	No Admission hyperglycemia	3 (1-6)/476	3 (1-5)/449	1.17 (0.93 - 1.46)	0.181	1.15 (0.91-1.45)	0.228	1.13 (0.96-1.33)	0.138	
Secondary outcomes										
mRS 0 to 4 at 90 days	Admission hyperglycemia	186/282 (66.0)	151/257 (58.8)	1.36 (0.96 - 1.93)	0.085	1.34 (0.91-1.98)	0.143	1.27 (0.99-1.63)	0.059	0.794
	No Admission hyperglycemia	332/476 (69.7)	336/449 (74.8)	1.29 (0.97 - 1.72)	0.085	1.27 (0.93-1.73)	0.139	1.24 (1.01-1.52)	0.040	
mRS 0 to 3 at 90 days	Admission hyperglycemia	148/282 (52.5)	115/257 (44.7)	1.36 (0.97 - 1.91)	0.073	1.31 (0.90-1.92)	0.158	1.25 (0.99-1.59)	0.065	0.754
	No Admission hyperglycemia	277/476 (58.2)	286/449 (63.7)	1.26 (0.97 - 1.64)	0.087	1.25 (0.93-1.68)	0.131	1.21 (1.01-1.46)	0.042	
mRS 0 to 2 at 90 days	Admission hyperglycemia	106/282 (37.6)	84/257 (32.7)	1.24 (0.87 - 1.77)	0.234	1.20 (0.81-1.77)	0.360	1.17 (0.91-1.50)	0.231	0.823
	No Admission hyperglycemia	210/476 (44.1)	214/449 (47.7)	1.15 (0.89 - 1.49)	0.280	1.16 (0.88-1.54)	0.298	1.12 (0.93-1.35)	0.220	
mRS 0 to 1 at 90 days	Admission hyperglycemia	56/282 (19.9)	53/257 (20.6)	0.95 (0.63 - 1.45)	0.825	0.86 (0.54-1.35)	0.509	0.88 (0.65-1.19)	0.402	0.586
	No Admission hyperglycemia	140/476 (29.4)	138/449 (30.7)	1.06 (0.80-1.41)	0.661	1.07 (0.79-0.45)	0.680	1.04 (0.85-1.27)	0.705	
NIHSS score at 5–7 days^c^	Admission hyperglycemia	13 (5-27)/283	14 (5-33)/256	-1.61 (-3.99 - 0.78)	0.186	-1.07(-3.17-1.03)	0.319	-1.11(-3.49-1.26)	0.358	0.832
	No Admission hyperglycemia	10 (3-22)/478	10 (3-20)/450	-0.99 (-2.71 - 0.73)	0.259	-0.91 (-2.50-0.69)	0.264	-0.87 (-2.59-0.84)	0.319	
	Admission hyperglycemia	33005/72448 (45.6)/256	36717/72448 (50.7)/283	1.11 (0.91 - 1.36)	0.304			WR:1.06 (0.87 - 1.30)	0.562	0.886
	No Admission hyperglycemia	98741/215100 (45.9)/478	107605/215100 (50.0)/450	1.09 (0.93 - 1.27)	0.276			WR:1.08 (0.92 - 1.26)	0.331	
Safety outcomes										
Mortality within 90 days	Admission hyperglycemia	77/282 (27.3)	89/257 (34.6)	0.71 (0.49 - 1.02)	0.066	0.73 (0.48-1.10)	0.131	0.78 (0.60-1.01)	0.055	0.615
	No Admission hyperglycemia	120/476 (25.2)	94/449 (20.9)	0.79 (0.58 - 1.07)	0.124	0.81 (0.58-1.13)	0.214	0.82 (0.66-1.02)	0.080	
sICH within 48 hours^d^	Admission hyperglycemia	26/277 (9.4)	31/251 (12.4)	0.74 (0.42 - 1.28)	0.274	0.75 (0.42-1.32)	0.314	0.76 (0.52-1.13)	0.172	0.918
	No Admission hyperglycemia	52/477 (10.9)	38/442 (8.6)	0.77 (0.50 - 1.19)	0.241	0.76 (0.49-1.19)	0.226	0.75 (0.55-1.03)	0.077	
Any ICH within 48 hours	Admission hyperglycemia	113/277 (40.8)	104/251 (41.4)	0.97 (0.69 - 1.38)	0.881	0.98 (0.69-1.40)	0.923	0.99 (0.77-1.26)	0.929	0.646
	No Admission hyperglycemia	171/477 (35.8)	166/442 (37.6)	1.08 (0.82 - 1.41)	0.592	1.08 (0.82-1.41)	0.598	1.07 (0.89-1.29)	0.484	

a Values adjusted for age, baseline NIHSS score, prestroke mRS score, baseline ASPECTS, use of intravenous thrombolysis, time from onset to randomization, and occlusion location.

b Common odds ratio; The common odds ratio was estimated from an ordinal logistic regression model. The mRS of functional disability ranges from 0 (no symptoms) to 6 (death). Data were not available for 1 patient in the admission hyperglycemia group and 5 patients in the no admission hyperglycemia group.

c The β values were estimated from a multivariable linear regression model. The win proportion was calculated as the number of wins for the methylprednisolone group over the control group in NIHSS scores among all possible pairs of NIHSS scores (taking 1 patient from the methylprednisolone group and 1 patient from the control group), divided by the total number of pairs. The results were adjusted using the inverse probability of treatment weighting method. NIHSS scores range from 0 to 42; higher scores indicate more severe neurological deficits.

d sICH was defined according to the Heidelberg bleeding classification (an increase in the NIHSS score of 4 points or an increase in the score for an NIHSS subcategory of 2 points with any intracranial hemorrhage on imaging results).

CI, confidence interval; ICH, intracranial hemorrhage; IPTW, inverse probability of treatment weighting; IQR, interquartile range; mRS, modified Rankin Scale; NIHSS, National Institutes of Health Stroke Scale; OR, odds ratio; sICH, symptomatic intracranial hemorrhage.

### Sensitivity analysis

Among patients with different blood glucose status, subgroup analyses were performed to evaluate whether the association between treatment and 90-day mRS score differed across various subgroups. No significant interactions were identified across subgroups stratified by age, sex, baseline ASPECTS, baseline NIHSS score, occlusion site, stroke etiology, the use of intravenous thrombolysis and time from onset to randomization (**Figure 2****;** **Supplementary Figure 6**-**8**). For patients with missing admission glucose, we performed a multiple-imputation sensitivity analysis, and the results were broadly consistent with those of the primary analysis (**Supplementary Table 3**).

**Figure 2:**
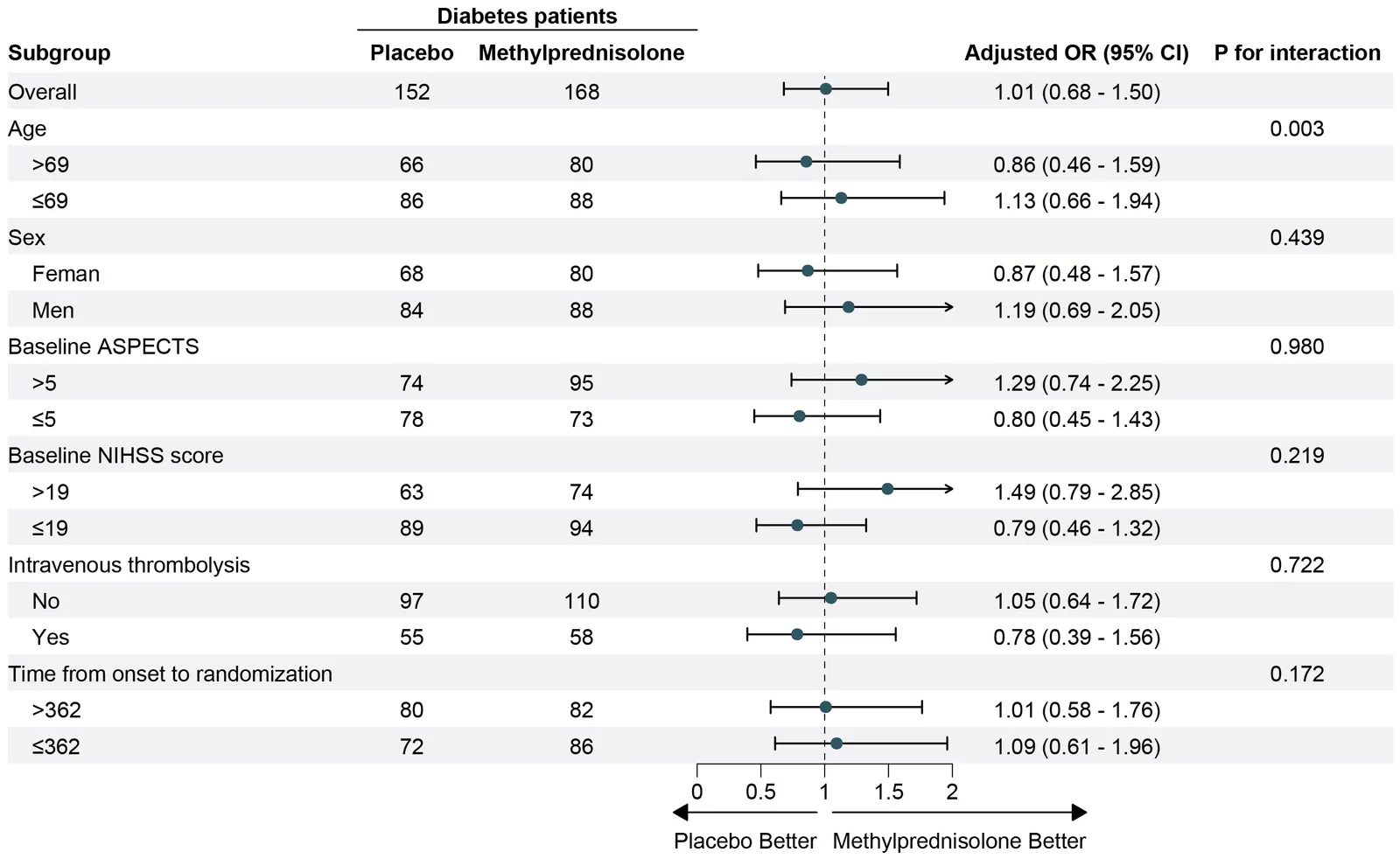
Subgroup analysis. The forest plot illustrates the differences in odds ratios for the ordinal modified Rankin Scale (mRS) score at 90 days across subgroups of patients with acute ischemic stroke due to the history of diabetes mellitus. Adjusted variables: age, baseline NIHSS score, prestroke mRS score, baseline ASPECTS, use of intravenous thrombolysis, time from stroke onset to randomization, and occlusion location. Abbreviations: ASPECTS, Alberta Stroke Program Early Computed Tomography Score; CI, confidence interval; NIHSS, National Institutes of Health Stroke Scale; OR, odds ratio.

### Margin effect

Considering that the same baseline glucose level may be observed in both patients with and without a history of diabetes, we analyzed the association between baseline glucose and clinical outcomes (an mRS score of 0–2 and mortality) among patients stratified by the history of diabetes (**Supplementary Figure 9**). The results demonstrated the predicted probability of an mRS score of 0–2 progressively decreased with increasing baseline glucose, while the predicted probability of mortality progressively increased in patients with or without diabetes mellitus.

## Discussion

In this secondary exploratory analysis of the MARVEL trial, we evaluated the effect of methylprednisolone as an adjunct to EVT in patients with and without diabetes mellitus, as well as between patients with and without admission hyperglycemia. The interaction between diabetes and treatment did not reach statistical significance. In patients without diabetes, a benefit trend was observed with adjunctive methylprednisolone. Moreover, in patients with diabetes, adjunctive use of methylprednisolone may be associated with a higher risk of any intracranial hemorrhage. In addition, the presence or absence of admission hyperglycemia did not significantly affect the effectiveness or safety of methylprednisolone.

Diabetes mellitus denotes a hyperglycemic state caused by various metabolic disorders, which feature either impaired insulin secretion, defective insulin action on target tissues, or frequently both (19). It has been previously established that a genetic predisposition to type 2 diabetes confers a higher risk of ischemic stroke, with particularly strong associations observed for large artery and small vessel stroke (20). Independent of hyperglycemia, a genetic tendency toward insulin resistance (but not β-cell dysfunction) correlated with an increased risk of large artery stroke, whereas a genetic predisposition involving both insulin resistance and β-cell dysfunction was linked to a higher risk of small vessel stroke (21). Acute hyperglycemia at admission was defined as a random plasma glucose level >140 mg/dL using the latest American Diabetes Association criteria (22). Among patients hospitalized for ischemic stroke, up to 40% present with elevated blood glucose levels, usually without a prior diagnosis of diabetes (23). Moreover, hyperglycemia is associated with a 3.3% increase in stroke-related mortality among non-diabetic individuals and correlates with more severe neurological impairments (24).

Our study revealed two principal findings regarding the primary outcome. First, among patients without a history of diabetes, adjunctive methylprednisolone to EVT may be associated with a trend toward favorable outcomes. In contrast, among patients with a history of diabetes, methylprednisolone did not demonstrate a similar trend toward benefit. This observation suggests that any potential benefit of methylprednisolone, may be attenuated or even negated by the presence of diabetes, thereby explaining the neutral result of the MARVEL trial, which showed no improvement in 90-day functional outcomes in the intention-to-treat population (8). Several mechanisms may account for this finding. Hyperglycemia in diabetic patients is associated with sustained low-grade inflammation, increased oxidative stress, and endothelial dysfunction (25, 26), which may render the anti-inflammatory effect of glucocorticoids less effective due to a “ceiling effect” of an already activated inflammatory cascade. Moreover, acute hyperglycemia at admission has been shown to exacerbate blood brain barrier disruption, promote mitochondrial dysfunction, and worsen reperfusion injury after EVT, pathways that are not readily reversed by methylprednisolone alone (27, 28).

Second, in both patients with and without admission hyperglycemia, no significant differences in outcomes were observed between the methylprednisolone and placebo groups. This result may be attributable to several potential explanations. Acute hyperglycemia exacerbates blood-brain barrier disruption, promotes mitochondrial dysfunction, and aggravates reperfusion injury, and these pathways cannot be readily reversed by methylprednisolone alone. Patients with admission hyperglycemia often already have a larger ischemic core volume and a higher core-to-penumbra ratio, suggesting that hyperglycemia itself may accelerate the conversion of the ischemic penumbra to the infarct core, thereby narrowing the window of “salvageable tissue” available for neuroprotective intervention (29–33). Admission hyperglycemia is independently associated with worse 90-day functional outcomes, higher mortality, and a higher risk of symptomatic intracranial hemorrhage. In such a population with an already poor baseline prognosis, the relative benefit potentially conferred by methylprednisolone may not be large enough in absolute terms to manifest statistically. The methylprednisolone regimen employed in this study may have been insufficient to meet the anti-inflammatory demands of patients with acute ischemic stroke, or may have failed to adequately cover the prolonged neuroinflammatory cascade that persists beyond the period of reperfusion. These mechanisms may collectively attenuate the neuroprotective anti-inflammatory effects of methylprednisolone, thereby rendering it less efficacious in this metabolically vulnerable population.

Moreover, in patients with diabetes, adjunctive methylprednisolone may be associated with a higher risk of any ICH, a phenomenon that can be jointly explained by the intrinsic cerebrovascular fragility of diabetes and the additional effects of glucocorticoids. In the diabetic state, cerebral microvessels already exhibit adverse structural remodeling, endothelial dysfunction, and impaired blood-brain barrier integrity, leaving the vascular bed inherently vulnerable (34). Meanwhile, diabetes can lead to persistently abnormal upregulation of vascular repair signals such as VEGF-R2, increasing blood-brain barrier permeability, and the direction of the response of these abnormalities to intervention may differ from that in the non-diabetic state (35). On this basis, methylprednisolone, through its well-established hyperglycemic effect, may further exacerbate glucose fluctuations (36), and both glycemic variability and admission hyperglycemia have been confirmed as independent predictors of symptomatic intracranial hemorrhage in patients after successful recanalization with EVT (37, 38). Therefore, in patients with diabetes, methylprednisolone may not only fail to effectively correct the diabetes-specific vascular signaling abnormalities, but may instead promote an increased risk of any ICH by aggravating metabolic disturbance and vascular fragility; whereas in patients without diabetes, this risk signal was not observed owing to the absence of this vulnerable background.

We note that the IPTW analysis yielded a statistically significant result (OR 1.16, 95% CI 1.02–1.33; *P* = 0.026) while the multivariable regression analysis did not (acOR 1.20, 95% CI 0.99–1.46; *P* = 0.061). We believe that this discrepancy may be attributable to several interrelated factors. First, the two methods target different estimates. IPTW estimates the marginal average treatment effect, whereas multivariable regression estimates the conditional treatment effect (39). For the odds ratio, a non-collapsible effect measure, these two quantities need not be numerically equal, particularly in the presence of effect modification. Second, within a subgroup of limited sample size, IPTW and regression adjustment, although asymptotically equivalent, may diverge in their finite-sample performance owing to differences in efficiency; in the present study, the narrower confidence interval yielded by the IPTW analysis suggests that weighting may have improved covariate balance within this subgroup.

## Limitation

The present study has some limitations. First, this was a secondary analysis of the MARVEL trial. The findings should be interpreted as exploratory and hypothesis generating. They do not supersede the original trial conclusions. Second, while the MARVEL trial randomization process was not stratified according to the history of diabetes mellitus or admission hyperglycemia, we used the IPTW method to control for confounders. Third, since the MARVEL trial enrolled only patients from China, the study results should be interpreted with caution. Fourth, in this study, the definition of diabetes status relied on medical history records, and the definition of hyperglycemia relied on a single admission glucose value. Dynamic glycemic parameters (such as glycemic variability or duration of hyperglycemia) were not incorporated, which may affect a comprehensive assessment of the relationship between glycemic-related factors and prognosis.

## Conclusion

In this exploratory secondary analysis of the MARVEL trial, the interaction between glycemia status and treatment did not reach statistical significance. A benefit trend was observed with adjunctive methylprednisolone in patients without diabetes. Moreover, adjunctive use of methylprednisolone in patients with diabetes may be with a higher risk of any intracranial hemorrhage. Admission hyperglycemia status did not significantly modify the effectiveness or safety of methylprednisolone. These findings warrant validation in further studies.

## Data Availability

The raw data supporting the conclusions of this article will be made available by the authors, without undue reservation.

## References

[1] Goyal M Menon BK van Zwam WH Dippel DW Mitchell PJ Demchuk AM . Endovascular thrombectomy after large-vessel ischaemic stroke: a meta-analysis of individual patient data from five randomised trials. Lancet (London England). (2016) 387:1723–31. doi: 10.1016/s0140-6736(16)00163-x 26898852

[2] Deng G Chu YH Xiao J Shang K Zhou LQ Qin C . Risk factors, pathophysiologic mechanisms, and potential treatment strategies of futile recanalization after endovascular therapy in acute ischemic stroke. Aging Dis. (2023) 14:2096–112. doi: 10.14336/AD.2023.0321-1 PMC10676786 37199580

[3] Xu J Wang A Meng X Yalkun G Xu A Gao Z . Edaravone dexborneol versus edaravone alone for the treatment of acute ischemic stroke: a phase III, randomized, double-blind, comparative trial. Stroke. (2021) 52:772–80. doi: 10.1161/strokeaha.120.031197 33588596

[4] Li J Meng X Shi FD Jing J Gu HQ Jin A . Colchicine in patients with acute ischaemic stroke or transient ischaemic attack (CHANCE-3): multicentre, double blind, randomised, placebo controlled trial. BMJ. (2024) 385:e079061. doi: 10.1136/bmj-2023-079061 38925803 PMC11200154

[5] Lu Y Guan L Wu J Yang Q Zhang M Zhou D . Efficacy and safety of minocycline in patients with acute ischaemic stroke (EMPHASIS): a multicentre, double-blind, randomised controlled trial. Lancet (London England). (2026) 407:679–88. doi: 10.1016/s0140-6736(25)01862-8 41628627

[6] Zheng C Li R Shen C Guo F Fan D Yang L . Methylprednisolone as adjunct to thrombectomy for acute intracranial internal carotid artery occlusion stroke: post hoc secondary analysis of the MARVEL randomized clinical trial. JAMA Netw Open. (2025) 8:e2459945. doi: 10.1001/jamanetworkopen.2024.59945 39964685 PMC11836765

[7] Ye Z Guo C Li Z Tian Z Li L Yang J . Adjunctive intravenous tirofiban plus methylprednisolone for acute ischemic stroke: a pooled analysis of the RESCUE BT and MARVEL trials. Med. (2026) 7:100990. doi: 10.1016/j.medj.2025.100990 41653926

[8] Yang Q Guo C Yue C Song J Yang J Investigators MTAftM . Methylprednisolone as adjunct to endovascular thrombectomy for large-vessel occlusion stroke: the MARVEL randomized clinical trial. JAMA. (2024) 331:840–9. doi: 10.1001/jama.2024.0626 PMC10853866 38329440

[9] Tamez-Pérez HE Quintanilla-Flores DL Rodríguez-Gutiérrez R González-González JG Tamez-Peña AL . Steroid hyperglycemia: prevalence, early detection and therapeutic recommendations: a narrative review. World J Diabetes. (2015) 6:1073–81. doi: 10.4239/wjd.v6.i8.1073 PMC4515447 26240704

[10] Lansang MC Hustak LK . Glucocorticoid-induced diabetes and adrenal suppression: how to detect and manage them. Cleveland Clinic J Med. (2011) 78:748–56. doi: 10.3949/ccjm.78a.10180 22049542

[11] Suh S Park MK . Glucocorticoid-induced diabetes mellitus: an important but overlooked problem. Endocrinol Metab (Seoul Korea). (2017) 32:180–9. doi: 10.3803/enm.2017.32.2.180 28555464 PMC5503862

[12] Rinkel LA Nguyen TTM Guglielmi V Groot AE Posthuma L Roos Y . High admission glucose is associated with poor outcome after endovascular treatment for ischemic stroke. Stroke. (2020) 51:3215–23. doi: 10.1161/strokeaha.120.029944 33054674

[13] Perez-Vega C Domingo RA Tripathi S Ramos-Fresnedo A Kashyap S Quinones-Hinojosa A . Influence of glucose levels on clinical outcome after mechanical thrombectomy for large-vessel occlusion: a systematic review and meta-analysis. J neurointerv Surg. (2022) 14:2397. doi: 10.1136/neurintsurg-2021-017771 34362794

[14] Yuen KC McDaniel PA Riddle MC . Twenty-four-hour profiles of plasma glucose, insulin, C-peptide and free fatty acid in subjects with varying degrees of glucose tolerance following short-term, medium-dose prednisone (20 mg/day) treatment: evidence for differing effects on insulin secretion and action. Clin Endocrinol. (2012) 77:224–32. doi: 10.1111/j.1365-2265.2011.04242.x 21973241 PMC3700529

[15] Vegiopoulos A Herzig S . Glucocorticoids, metabolism and metabolic diseases. Mol Cell Endocrinol. (2007) 275:43–61. doi: 10.1016/j.mce.2007.05.015 17624658

[16] Ye Z Guo C Li Z Tian Z Li L Yang J . Adjunctive intravenous tirofiban plus methylprednisolone for acute ischemic stroke: a pooled analysis of the RESCUE BT and MARVEL trials. Med. (2026) 7:100990. doi: 10.1016/j.medj.2025.100990 41653926

[17] Guo C Cavalcante F Yang S Suzuki K Treurniet K Kaesmacher J . Stroke etiologies with intravenous thrombolysis before thrombectomy and functional outcomes in anterior circulation large vessel occlusion. Ann Neurol. (2026) 100:180–90. doi: 10.1002/ana.78225 42026777

[18] Moons KG Donders RA Stijnen T Harrell FE Jr. Using the outcome for imputation of missing predictor values was preferred. J Clin Epidemiol. (2006) 59:1092–101. doi: 10.1016/j.jclinepi.2006.01.009 16980150

[19] Colagiuri S . Definition and classification of diabetes and prediabetes and emerging data on phenotypes. Endocrinol Metab Clinics North America. (2021) 50:319–36. doi: 10.1016/j.ecl.2021.06.004 34399948

[20] Strawbridge RJ Dupuis J Prokopenko I Barker A Ahlqvist E Rybin D . Genome-wide association identifies nine common variants associated with fasting proinsulin levels and provides new insights into the pathophysiology of type 2 diabetes. Diabetes. (2011) 60:2624–34. doi: 10.2337/db11-0415 21873549 PMC3178302

[21] Georgakis MK Harshfield EL Malik R Franceschini N Langenberg C Wareham NJ . Diabetes mellitus, glycemic traits, and cerebrovascular disease: a Mendelian randomization study. Neurology. (2021) 96:e1732–42. doi: 10.1212/wnl.0000000000011555 33495378 PMC8055310

[22] Masrur S Cox M Bhatt DL Smith EE Ellrodt G Fonarow GC . Association of acute and chronic hyperglycemia with acute ischemic stroke outcomes post-thrombolysis: findings from Get With The Guidelines-Stroke. J Am Heart Assoc. (2015) 4:e002193. doi: 10.1161/jaha.115.002193 26408015 PMC4845108

[23] Liu H Jin A Pan Y Meng X Li H Li Z . Efficacy and safety of intravenous tenecteplase versus alteplase in treating acute ischemic stroke with diabetes and admission hyperglycemia. J Am Heart Assoc. (2024) 13:e036393. doi: 10.1161/jaha.124.036393 39392168 PMC11935594

[24] Capes SE Hunt D Malmberg K Pathak P Gerstein HC . Stress hyperglycemia and prognosis of stroke in nondiabetic and diabetic patients: a systematic overview. Stroke. (2001) 32:2426–32. doi: 10.1161/hs1001.096194 11588337

[25] Katakami N . Mechanism of development of atherosclerosis and cardiovascular disease in diabetes mellitus. J Atheroscl Thromb. (2018) 25:27–39. doi: 10.5551/jat.rv17014 28966336 PMC5770221

[26] Rösen P Nawroth PP King G Möller W Tritschler HJ Packer L . The role of oxidative stress in the onset and progression of diabetes and its complications: a summary of a Congress Series sponsored by UNESCO-MCBN, the American Diabetes Association and the German Diabetes Society. Diabetes/Metabolism Res Rev. (2001) 17:189–212. doi: 10.1002/dmrr.196 11424232

[27] Song G Liu X Lu Z Guan J Chen X Li Y . Relationship between stress hyperglycaemic ratio (SHR) and critical illness: a systematic review. Cardiovasc Diabetol. (2025) 24:188. doi: 10.1186/s12933-025-02751-3 40317019 PMC12049067

[28] Kim JT Lee JS Kim BJ Kang J Lee KJ Park JM . Admission hyperglycemia, stroke subtypes, outcomes in acute ischemic stroke. Diabetes Res Clin Pract. (2023) 196:110257. doi: 10.1016/j.diabres.2023.110257 36642337

[29] Zhang Y Yin X Liu T Ji W Wang G . Association between the stress hyperglycemia ratio and mortality in patients with acute ischemic stroke. Sci Rep. (2024) 14:20962. doi: 10.1038/s41598-024-71778-5 39251650 PMC11385565

[30] Muscari A Falcone R Recinella G Faccioli L Forti P Pastore Trossello M . Prognostic significance of diabetes and stress hyperglycemia in acute stroke patients. Diabetol Metab Syndrome. (2022) 14:126. doi: 10.1186/s13098-022-00896-9 36038896 PMC9422130

[31] Zhang L Gao F Tian Y Li L Tian Y Guo C . Association between admission hyperglycemia and outcomes after endovascular treatment in acute basilar artery occlusion. Neurol Ther. (2023) 12:1285–97. doi: 10.1007/s40120-023-00502-8 37266794 PMC10310590

[32] MacIntosh BJ Cohen E Colby-Milley J Fang J Zhou L Ouk M . Diabetes mellitus is associated with poor in-hospital and long-term outcomes in young and midlife stroke survivors. J Am Heart Assoc. (2021) 10:e019991. doi: 10.1161/jaha.120.019991 34219470 PMC8483482

[33] Maida CD Daidone M Pacinella G Norrito RL Pinto A Tuttolomondo A . Diabetes and ischemic stroke: an old and new relationship an overview of the close interaction between these diseases. Int J Mol Sci. (2022) 14:neurintsurg-2021–01777. doi: 10.3390/ijms23042397 35216512 PMC8877605

[34] Zhang Q Liu J Ji X . Diabetic cerebral microvascular disorder: from key pathologies to targeted interventions. Ann Med. (2026) 58:2654248. doi: 10.1080/07853890.2026.2654248 42046408 PMC13126952

[35] Reeson P Tennant KA Gerrow K Wang J Weiser Novak S Thompson K . Delayed inhibition of VEGF signaling after stroke attenuates blood-brain barrier breakdown and improves functional recovery in a comorbidity-dependent manner. J Neuroscience: Off J Soc For Neurosci. (2015) 35:5128–43. doi: 10.1523/jneurosci.2810-14.2015 25834040 PMC6705411

[36] van Raalte DH Diamant M Ouwens DM Ijzerman RG Linssen MM Guigas B . Glucocorticoid treatment impairs microvascular function in healthy men in association with its adverse effects on glucose metabolism and blood pressure: a randomised controlled trial. Diabetologia. (2013) 56:2383–91. doi: 10.1007/s00125-013-3016-8 23933953

[37] Kim TJ Lee JS Park SH Ko SB . Short-term glycemic variability and hemorrhagic transformation after successful endovascular thrombectomy. Trans Stroke Res. (2021) 12:968–75. doi: 10.1007/s12975-021-00895-4 33576937

[38] Hao Z Yang C Xiang L Wu B Liu M . Risk factors for intracranial hemorrhage after mechanical thrombectomy: a systematic review and meta-analysis. Expert Rev Neurother. (2019) 19:927–35. doi: 10.1080/14737175.2019.1632191 31200607

[39] Phillippo DM Remiro-Azócar A Heath A Baio G Dias S Ades AE . Effect modification and non-collapsibility together may lead to conflicting treatment decisions: a review of marginal and conditional estimands and recommendations for decision-making. Res Synth Methods. (2025) 16:323–49. doi: 10.1017/rsm.2025.2 41626969 PMC12527544

