# Supplementary material for: Outcomes of methylprednisolone as an adjunct to endovascular therapy in patients with diabetes and admission hyperglycemia
Source: Front Endocrinol (Lausanne). 2026 Oct 8;17:1932813. doi: 10.3389/fendo.2026.1932813 (PMC13686846; doi:10.3389/fendo.2026.1932813)
Supplement: Supplementary file 1 [file DataSheet1.pdf]

Supplemental material

Supplementary methods 1. Inverse probability of treatment weighting.

Supplementary methods 2. Handling of missing values.

Supplementary Table 1. Baseline Characteristics of Methylprednisolone Versus Placebo Stratified by Diabetes History after Inverse Probability of Treatment Weighting.

Supplementary Table 2. Baseline Characteristics of Methylprednisolone Versus Placebo Stratified by Admission Hyperglycemia Inverse Probability of Treatment Weighting.

Supplementary Table 3. Outcomes according to the treatment in patients with and without admission hyperglycemia after multiple imputation.

Supplementary Figure 1. Flow Chart of Study Design and Patient Selection.

Supplementary Figure 2. Inverse probability of treatment weighting for patients with diabetes.

Supplementary Figure 3. Inverse probability of treatment weighting for patients without diabetes.

Supplementary Figure 4. Inverse probability of treatment weighting for patients with admission hyperglycemia.

Supplementary Figure 5. Inverse probability of treatment weighting for patients without admission hyperglycemia.

Supplement Figure 6. Subgroup analysis of efficacy between methylprednisolone and

placebo groups in patients with admission hyperglycemia.

Supplement Figure 7. Subgroup analysis of efficacy between methylprednisolone and placebo groups in patients without diabetes.

Supplement Figure 8. Subgroup analysis of efficacy between methylprednisolone and placebo groups in patients without admission hyperglycemia.

Supplement Figure 9. Margin effect.

Supplementary method 1. Inverse Probability of Treatment Weighting

Inverse probability of treatment weighting (IPTW) was applied as a sensitivity analysis for the randomized comparison of methylprednisolone versus placebo within each glycemic subgroup. The rationale for using IPTW in this context was not to control for confounding by indication, since randomization already balances measured and unmeasured confounders in expectation at the trial level.

Selection of covariates. The propensity-score model included seven covariates: age, baseline NIHSS score, pre-stroke mRS score, baseline ASPECTS, use of intravenous thrombolysis, time from stroke onset to randomization, and occlusion location. Their selection was based on established subject-matter knowledge and clinical relevance, reflecting well-established prognostic factors for functional outcome after endovascular therapy in acute ischemic stroke.

Calculation of weights. We employed the standard inverse probability of treatment weighting approach targeting the average treatment effect (ATE). Each patient received a weight equal to the inverse of the estimated propensity of receiving the treatment actually received. Specifically, for patients in the methylprednisolone group, the weight was $1/e(X)$, and for patients in the placebo group, the weight was $1/(1-e(X))$, where $e(X)$ represents the estimated propensity score. Stabilized weights were not used in the primary analysis.

Handling of extreme weights. No weight truncation or trimming was applied in the primary IPTW analysis.

Variance estimation. Robust sandwich variance estimators were used to account for the weighted nature of the synthetic sample and to appropriately reflect the uncertainty introduced by propensity-score estimation. This approach is consistent with best practice recommendations for IPTW analyses and has been shown to provide accurate standard errors and confidence intervals.

Supplementary methods 2. Handling of missing values.

We applied sample imputation method for the covariate ASPECT score to perform IPTW. Baseline ASPECT score was continuous variables. For continuous variables, missing values were imputed using a kernel density estimation approach. The probability density function was estimated from the non-missing values, and missing values were subsequently replaced by random samples drawn from the estimated distribution. We set a seed in our R code before performing the imputation to ensure reproducibility.

Supplementary Table 1. Baseline Characteristics of Methylprednisolone Versus Placebo Stratified by Diabetes History after Inverse Probability of Treatment Weighting.

**Characteristic**	**Diabetes (N= 641.876)**				**Without Diabetes (N= 2713.990)**			
	**Methylprednisolone (N= 320.563)**	**Placebo (N= 321.313)**	**SMD**	***P* value**	**Methylprednisolone (N=1356.032)**	**Placebo (N=1357.958)**	**SMD**	***P* value**
Age, median (IQR), y	68 (62-75)	69 (59-77)	0.003	0.903	69 (59-76)	69 (58-77)	0.001	0.912
Sex, man, No. (%)	166.3 (51.9)	175.2 (54.5)	0.053	0.637	784.7 (57.9)	777.9 (57.3)	0.012	0.828
Medical history[a]								
Hypertension	251.5 (78.4)	241.1 (75.0)	0.081	0.476	759.2 (56.0)	797.4 (58.7)	0.055	0.314
Atrial fibrillation	129.6 (40.4)	119.6 (37.2)	0.066	0.559	584.1 (43.1)	568.0 (41.8)	0.025	0.644
Hyperlipidemia	125.5 (39.2)	109.0 (33.9)	0.109	0.339	399.1 (29.4)	389.9 (28.7)	0.016	0.774
Coronary heart disease	85.0 (26.5)	67.5 (21.0)	0.130	0.254	217.1 (16.0)	258.7 (19.0)	0.080	0.145
Cerebral ischemia	56.5 (17.6)	52.3 (16.3)	0.035	0.753	215.0 (15.9)	186.9 (13.8)	0.059	0.288
Valvular heart disease	24.9 (7.8)	36.7 (11.4)	0.124	0.271	198.1 (14.6)	196.1 (14.4)	0.005	0.932
Intracranial hemorrhage	7.5 (2.3)	1.6 (0.5)	0.155	0.132	14.5 (1.1)	18.1 (1.3)	0.024	0.649
Transient ischemic attack	3.8 (1.2)	0.0 (0.0)	0.155	0.162	8.7 (0.6)	7.5 (0.5)	0.012	0.821
Smoking (current)	95.3 (29.7)	66.6 (20.7)	0.208	0.068	366.9 (27.1)	420.9 (31.0)	0.087	0.112
Prestroke modified Rankin Scale score 0-4[b]	21.0 (6.5)	14.8 (4.6)	0.084	0.438	44.6 (3.3)	39.0 (2.9)	0.024	0.675
Glucose level at hospital arrival, median (IQR), mmol/L	10.9 (8.2-14.4)	10.4 (8.2-14.0)	0.027	0.591	6.9 (6.1-8.1)	6.7 (5.8-7.8)	0.104	0.023

Baseline NIHSS score, median (IQR)[c]	18 (16-21)	19 (16-21)	0.001	0.886	19 (16-21)	19 (16-21)	<0.001	0.901
Baseline ASPECTS, median (IQR)[d]	6 (4-7)	6 (4-8)	0.002	0.965	6 (4-7)	5 (4-7)	<0.001	0.902
Stroke etiology[e]			0.138	0.678			0.147	0.067
Large artery atherosclerosis	156.2 (48.7)	171.6 (53.4)			437.4 (32.3)	508.4 (37.4)		
Cardioembolic	132.3 (41.3)	111.7 (34.8)			724.3 (53.4)	658.7 (48.5)		
Others	7.7 (2.4)	8.0 (2.5)			55.1 (4.1)	76.7 (5.7)		
Unknown	24.3 (7.6)	30.0 (9.3)			139.2 (10.3)	114.1 (8.4)		
Occlusion site			0.003	1.000			0.001	1.000
ICA	106.4 (33.2)	107.2 (33.4)			474.9 (35.0)	475.2 (35.0)		
M1	167.8 (52.3)	167.8 (52.2)			706.8 (52.1)	707.9 (52.1)		
M2	46.4 (14.5)	46.3 (14.4)			174.3 (12.9)	174.9 (12.9)		
Intravenous thrombolysis	112.0 (34.9)	111.6 (34.7)	0.004	0.969	510.9 (37.7)	511.8 (37.7)	<0.001	0.995
Time from last known well, median (IQR), min								
To puncture	344 (223-555)	111.6 (34.7)	0.027	0.304	342 (234-621)	365 (240-593)	0.009	0.693
To randomization	358 (235-579)	384 (243-621)	0.007	0.242	351 (236-629)	370 (241-590)	0.001	0.916
To recanalization	439 (301-644)	384 (248-615)	0.016	0.370	423 (314-704)	446 (313-697)	0.016	0.652
Time from randomization to initial treatment, median (IQR), h	8 (6-13)	461 (316-707)	0.108	0.686	8 (6-14)	8 (6-13)	0.092	0.368
Blood pressure at hospital arrival, median (IQR), mm Hg								
Systolic	148 (130-165)	150 (132-162)	0.006	0.790	141 (126-158)	143 (125-160)	0.009	0.662
Diastolic	84 (75-95)	86 (75-94)	0.050	0.522	83 (74-92)	83 (74-94)	0.057	0.291

[a] Comorbidities based on family or patient report.

[b] Scores on the mRS of functional disability range from 0 (no symptoms) to 6 (death).

[c] Scores on the NIHSS range from 0 to 42, with higher scores indicating more severe neurologic deficit.

[d] The ASPECTS is an imaging measure of the extent of ischemic stroke. Scores range from 0 to 10, with higher scores indicating a smaller infarct core. Listed are values for the core laboratory assessment.

[e] Stroke etiology was categorized according to the Trial of ORG 10172 in Acute Stroke Treatment (TOAST) classification.

Abbreviations: ASPECTS, Alberta Stroke Program Early CT Score; ICA, Intracranial internal carotid artery; IQR, Interquartile range; M1, M1 middle cerebral artery segment; M2, M2 middle cerebral artery segment; mRS, modified Rankin Scale; NIHSS, National Institutes of Health Stroke Scale; SMD, Standardized mean differences; TOAST, Trial of ORG 10172 in Acute Stroke Treatment.

Supplementary Table 2. Baseline Characteristics of Methylprednisolone Versus Placebo Stratified by Admission Hyperglycemia Inverse Probability of Treatment Weighting.

Characteristic	Admission hyperglycemia (N=1079.862)				No admission hyperglycemia (N= 1859.990)			
	Methylprednisolone (N= 539.642)	Placebo (N= 540.220)	SMD	*P* value	Methylprednisolone (N= 929.677)	Placebo (N= 930.312)	SMD	*P* value
Age, median (IQR), y	70 (62-76)	70 (59-77)	0.001	0.816	68 (58-76)	68 (58-76)	<0.001	0.978
Sex, man, No. (%)	264.9 (49.1)	265.0 (49.1)	<0.001	0.996	559.8 (60.2)	580.5 (62.4)	0.045	0.495
Medical history[a]								
Hypertension	368.7 (68.3)	397.1 (73.5)	0.114	0.190	530.6 (57.1)	546.5 (58.7)	0.034	0.608
Atrial fibrillation	234.4 (43.4)	248.9 (46.1)	0.053	0.540	392.3 (42.2)	361.1 (38.8)	0.069	0.297
Hyperlipidemia	219.6 (40.7)	230.1 (42.6)	0.038	0.659	58.1 (6.3)	56.7 (6.1)	0.007	0.922
Diabetes	185.9 (34.4)	170.0 (31.5)	0.063	0.467	292.9 (31.5)	277.8 (29.9)	0.036	0.588
Coronary heart disease	122.5 (22.7)	127.5 (23.6)	0.021	0.806	141.5 (15.2)	160.6 (17.3)	0.056	0.402
Cerebral ischemia	79.2 (14.7)	83.0 (15.4)	0.019	0.823	163.7 (17.6)	124.3 (13.4)	0.118	0.077
Valvular heart disease	62.5 (11.6)	58.5 (10.8)	0.024	0.781	125.2 (13.5)	144.7 (15.6)	0.059	0.370
Intracranial hemorrhage	9.4 (1.7)	8.5 (1.6)	0.013	0.877	13.7 (1.5)	7.9 (0.9)	0.058	0.376
Transient ischemic attack	3.8 (0.7)	0.0 (0.0)	0.119	0.159	6.3 (0.7)	7.7 (0.8)	0.018	0.782
Smoking (current)	122.4 (22.7)	120.3 (22.3)	0.010	0.910	276.3 (29.7)	288.1 (31.0)	0.027	0.680
Prestroke modified Rankin Scale score 0-4[b]	24.9 (4.6)	18.7 (3.5)	0.059	0.513	31.7 (3.4)	29.4 (3.2)	0.014	0.837
Glucose level at hospital arrival, median (IQR), mmol/L	9.5 (8.6-12.1)	9.8 (8.5-12.1)	0.018	0.788	6.4 (5.8-7.0)	6.3 (5.6-7.0)	0.122	0.111

Baseline NIHSS score, median (IQR)[c]	19 (16-21)	19 (17-21)	0.004	0.945	19 (17-21)	19 (16-21)	0.001	0.810
Baseline ASPECTS, median (IQR)[d]	5 (4-7)	5 (4-7)	0.001	0.948	6 (4-7)	6 (4-7)	0.001	0.924
Stroke etiology[e]			0.110	0.656			0.159	0.122
Large artery atherosclerosis	202.2 (37.5)	231.0 (42.8)			310.1 (33.4)	368.6 (39.6)		
Cardioembolic	271.8 (50.4)	246.3 (45.6)			479.9 (51.6)	433.1 (46.6)		
Others	23.3 (4.3)	21.2 (3.9)			34.2 (3.7)	45.5 (4.9)		
Unknown	42.3 (7.8)	41.7 (7.7)			105.4 (11.3)	83.1 (8.9)		
Occlusion site[f]			0.003	0.999			<0.001	1.000
ICA	188.9 (35.0)	188.8 (35.0)			318.0 (34.2)	318.1 (34.2)		
M1	284.8 (52.8)	284.8 (52.7)			486.0 (52.3)	486.4 (52.3)		
M2	66.0 (12.2)	66.5 (12.3)			125.7 (13.5)	125.8 (13.5)		
Intravenous thrombolysis	205.8 (38.1)	205.8 (38.1)	0.001	0.993	338.5 (36.4)	338.7 (36.4)	<0.001	0.998
Time from last known well, median (IQR), min								
To puncture	316 (225-540)	350 (240-562)	0.003	0.208	363 (246-660)	383 (244-627)	0.002	0.838
To randomization	329 (224-549)	360 (238-586)	<0.001	0.462	369 (251-696)	385 (255-617)	0.001	0.944
To recanalization	412 (303-621)	430 (308-660)	0.024	0.576	441 (320-745)	472 (325-745)	0.017	0.876
Time from randomization to initial treatment, median (IQR), h	8 (6-13)	8 (6-14)	0.076	0.169	8 (6-14)	8 (6-13)	0.125	0.100
Blood pressure at hospital arrival, median (IQR), mm Hg								
Systolic	148 (132-165)	148 (130-162)	0.016	0.945	140 (125-157)	143 (125-160)	0.036	0.384
Diastolic	82 (75-95)	85 (75-96)	0.106	0.275	84 (73-92)	84 (75-94)	0.057	0.300

[a] Comorbidities based on family or patient report.

[b] Scores on the mRS of functional disability range from 0 (no symptoms) to 6 (death).

[c] Scores on the NIHSS range from 0 to 42, with higher scores indicating more severe neurologic deficit.

[d] The ASPECTS is an imaging measure of the extent of ischemic stroke. Scores range from 0 to 10, with higher scores indicating a smaller infarct core. Listed are values for the core laboratory assessment.

[e] Stroke etiology was categorized according to the Trial of ORG 10172 in Acute Stroke Treatment (TOAST) classification.

[f] One patient had a basilar artery occlusion and 1 patient had an anterior cerebral artery occlusion in the methylprednisolone group.

Abbreviations: ASPECTS, Alberta Stroke Program Early CT Score; ICA, Intracranial internal carotid artery; IQR, Interquartile range; M1, M1 middle cerebral artery segment; M2, M2 middle cerebral artery segment; mRS, modified Rankin Scale; NIHSS, National Institutes of Health Stroke Scale; SMD, Standardized mean differences; TOAST, Trial of ORG 10172 in Acute Stroke Treatment.

Supplementary Table 3. Outcomes according to the treatment in patients with and without admission hyperglycemia after multiple imputation

Outcomes		Methylprednisolone (N=841)	Placebo (N=837)	Unadjusted OR (95% CI)	P value	Adjusted OR (95% CI) [a]	*P* value	IPTW adjusted Value (95% CI) [a]	*P* value
**Primary outcome**									
mRS score at 90 days[b]	Admission hyperglycemia	4 (2 - 6)/298	3 (2 - 6)/328	1.25 (0.93 - 1.68)	0.131	1.19 (0.88 - 1.61)	0.267	1.18 (0.95 - 1.46)	0.144
	No Admission hyperglycemia	3 (1 - 6)/543	3 (1 - 5)/509	1.13 (0.91 - 1.42)	0.272	1.14 (0.90 - 1.43)	0.268	1.11 (0.94 - 1.31)	0.214
**Secondary outcomes**									
mRS 0 to 4 at 90 days	Admission hyperglycemia	175/298 (58.7)	215/328 (65.5)	1.35 (0.96 - 1.90)	0.089	1.32 (0.90 - 1.94)	0.153	1.26 (0.98 - 1.63)	0.071
	No Admission hyperglycemia	382/543 (70.3)	382/509 (75.0)	1.27 (0.95 - 1.69)	0.105	1.27 (0.93 - 1.74)	0.125	1.24 (1.00 - 1.53)	0.047
mRS 0 to 3 at 90 days	Admission hyperglycemia	136/298 (45.6)	172/328 (52.4)	1.31 (0.94 - 1.83)	0.111	1.27 (0.87 - 1.85)	0.215	1.22 (0.95 - 1.57)	0.112
	No Admission hyperglycemia	324/543 (59.7)	324/509 (63.7)	1.18 (0.91 - 1.53)	0.211	1.18 (0.88 - 1.58)	0.259	1.15 (0.95 - 1.40)	0.152
mRS 0 to 2 at 90 days	Admission hyperglycemia	100/298 (33.6)	124/328 (37.8)	1.22 (0.86 - 1.72)	0.261	1.18 (0.81 - 1.73)	0.381	1.16 (0.90 - 1.50)	0.247
	No Admission hyperglycemia	245/543 (45.1)	243/509 (47.7)	1.11 (0.86 - 1.43)	0.416	1.13 (0.86 - 1.49)	0.386	1.10 (0.91 - 1.32)	0.331
mRS 0 to 1	Admission	62/298 (20.8)	66/328	0.96 (0.64 -	0.844	0.88 (0.57 -	0.583	0.90 (0.67 - 1.22)	0.509

at 90 days	hyperglycemia		(20.1)	1.44)		1.37)			
	No Admission hyperglycemia	159/543 (29.3)	154/509 (30.3)	1.05 (0.80 - 1.37)	0.749	1.06 (0.79 - 1.42)	0.719	1.03 (0.84 - 1.25)	0.778
NIHSS score at 5-7 days[c]	Admission hyperglycemia	13 (5 - 33)/298	12 (5 - 27)/328	-1.54 (-3.90 - 0.81)	0.199	-1.13 (-3.26 - 1.00)	0.298	-1.13 (-3.50 - 1.24)	0.348
	No Admission hyperglycemia	10 (3 - 22)/543	10 (3 - 20)/509	-0.95 (-2.63 - 0.74)	0.270	-0.98 (-2.55 - 0.58)	0.219	-0.96 (-2.65 - 0.72)	0.261
**Safety outcomes**									
Mortality within 90 days	Admission hyperglycemia	102/298 (34.2)	87/328 (26.5)	0.70 (0.48 - 1.00)	0.051	0.71 (0.47 - 1.06)	0.095	0.75 (0.57 - 0.98)	0.038
	No Admission hyperglycemia	138/543 (25.4)	108/509 (21.2)	0.79 (0.58 - 1.07)	0.124	0.79 (0.57 - 1.10)	0.166	0.82 (0.65 - 1.02)	0.073
sICH within 48 hours	Admission hyperglycemia	40/298 (13.4)	30/328 (9.1)	0.66 (0.38 - 1.13)	0.125	0.67 (0.38 - 1.17)	0.161	0.68 (0.45 - 1.03)	0.071
	No Admission hyperglycemia	62/543 (11.4)	46/509 (9.0)	0.77 (0.51 - 1.16)	0.214	0.76 (0.50 - 1.15)	0.188	0.75 (0.56 - 1.01)	0.062
Any ICH within 48 hours	Admission hyperglycemia	120/298 (40.3)	130/328 (39.6)	0.97 (0.68 - 1.37)	0.845	0.98 (0.68 - 1.40)	0.902	0.98 (0.75 - 1.28)	0.884
	No Admission hyperglycemia	195/543 (35.9)	194/509 (38.1)	1.10 (0.85 - 1.42)	0.480	1.09 (0.84 - 1.42)	0.519	1.09 (0.90 - 1.32)	0.382

[a] Values adjusted for age, baseline NIHSS score, prestroke mRS score, baseline ASPECTS, use of intravenous thrombolysis, time from onset to randomization, and occlusion location.

[b] Common odds ratio; The common odds ratio was estimated from an ordinal logistic regression model. The mRS of functional disability ranges from 0 (no symptoms) to 6 (death).

[c] The β values were estimated from a multivariable linear regression model. The win proportion was calculated as the number of wins for the methylprednisolone group over the control group in NIHSS scores among all possible pairs of NIHSS scores (taking 1 patient from the methylprednisolone group and 1 patient from the control group), divided by the total number of pairs. The results were adjusted using the inverse probability of treatment weighting method. NIHSS scores range from 0 to 42; higher scores indicate more severe neurological deficits.

Abbreviations: CI, confidence interval; ICH, intracranial hemorrhage; IPTW, inverse probability of treatment weighting; IQR, interquartile range; mRS, modified Rankin Scale; NIHSS, National Institutes of Health Stroke Scale; OR, odds ratio; sICH, symptomatic intracranial hemorrhage.

Supplementary Figure 1. Flow Chart of Study Design and Patient Selection.

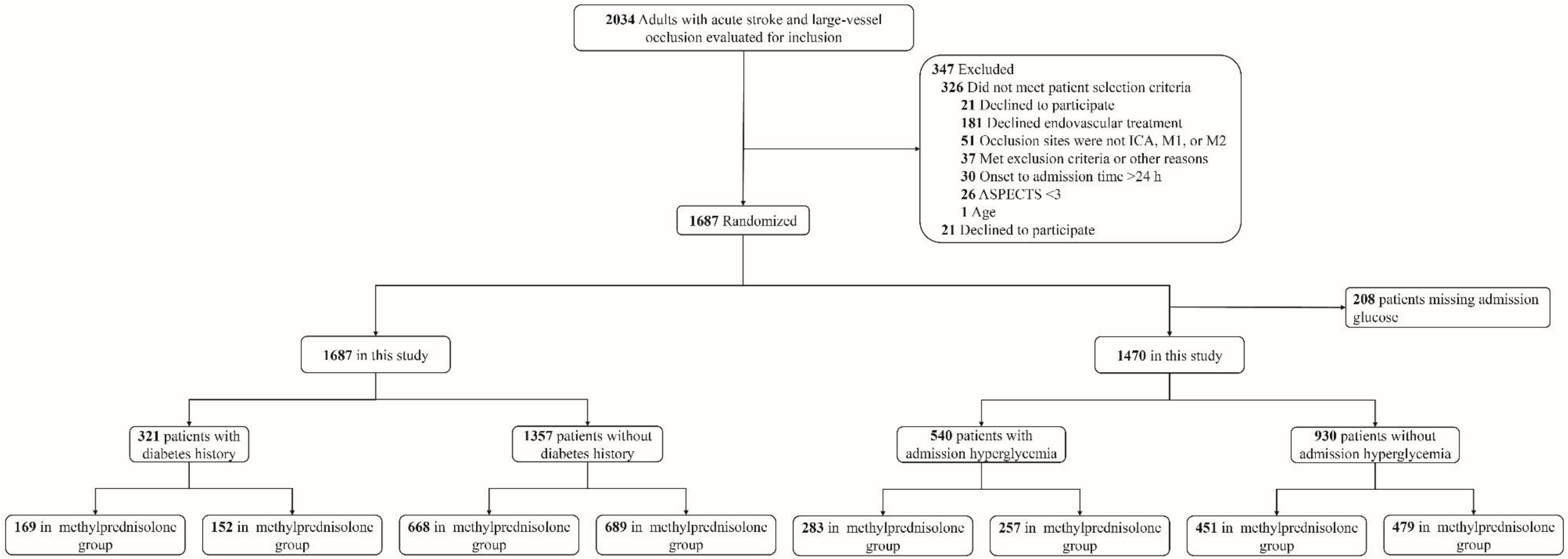

Supplementary Figure 2. Inverse probability of treatment weighting for patients with diabetes.

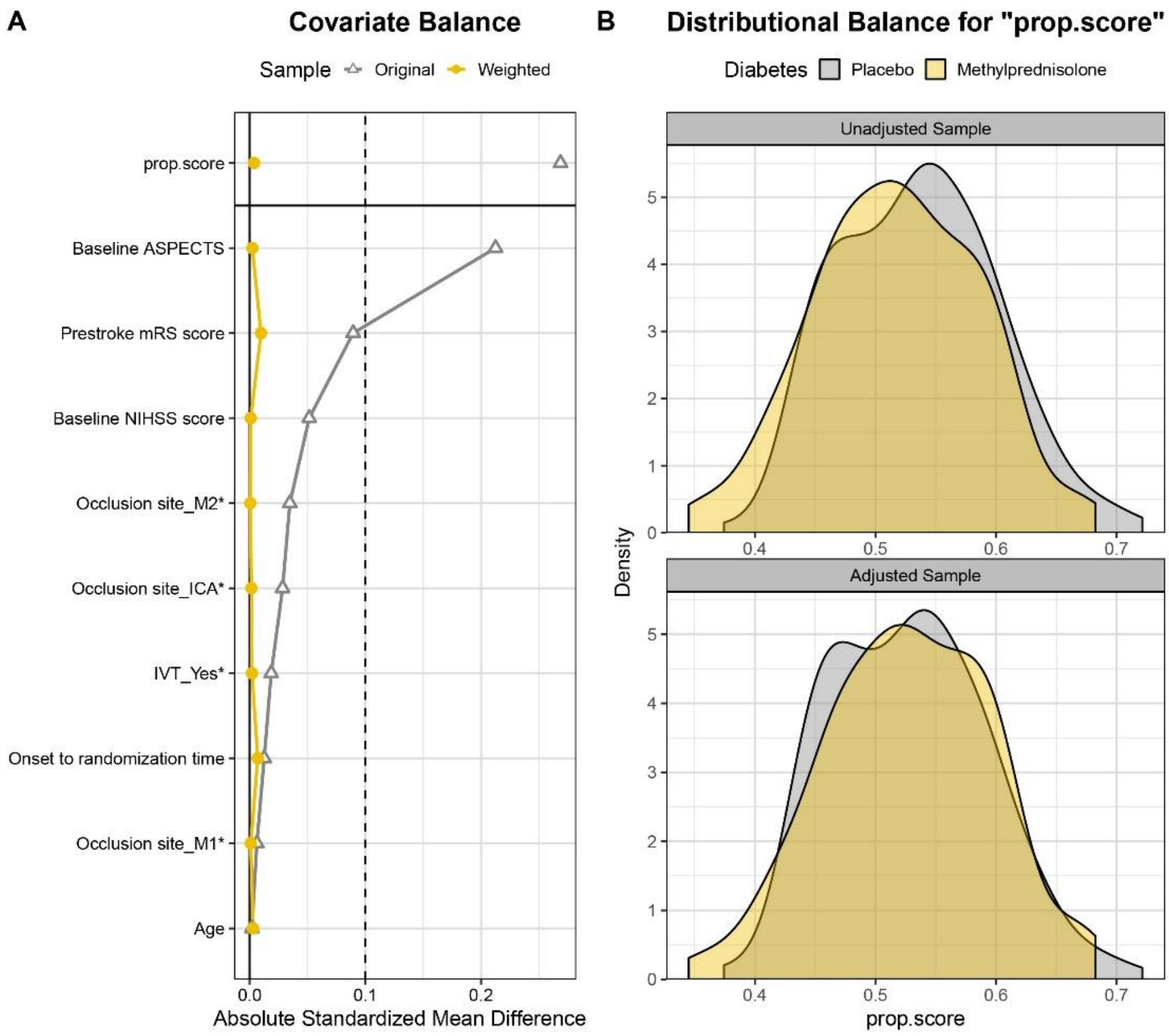

(A) Standardized mean differences before inverse probability of treatment weighting (gray) and after inverse probability of treatment weighting (yellow) for all selected baseline covariates. (B) Distributions of propensity scores before and after inverse probability of treatment weighting for the Methylprednisolone and control group.

Abbreviations: ASPECTS, Alberta Stroke Program Early Computed Tomography Score; EVT, endovascular treatment; ICA, internal carotid artery; IVT, intravenous thrombolysis; mRS, modified Rankin Scale; NIHSS, National Institutes of Health Stroke Scale.

Supplementary Figure 3. Inverse probability of treatment weighting for patients without diabetes.

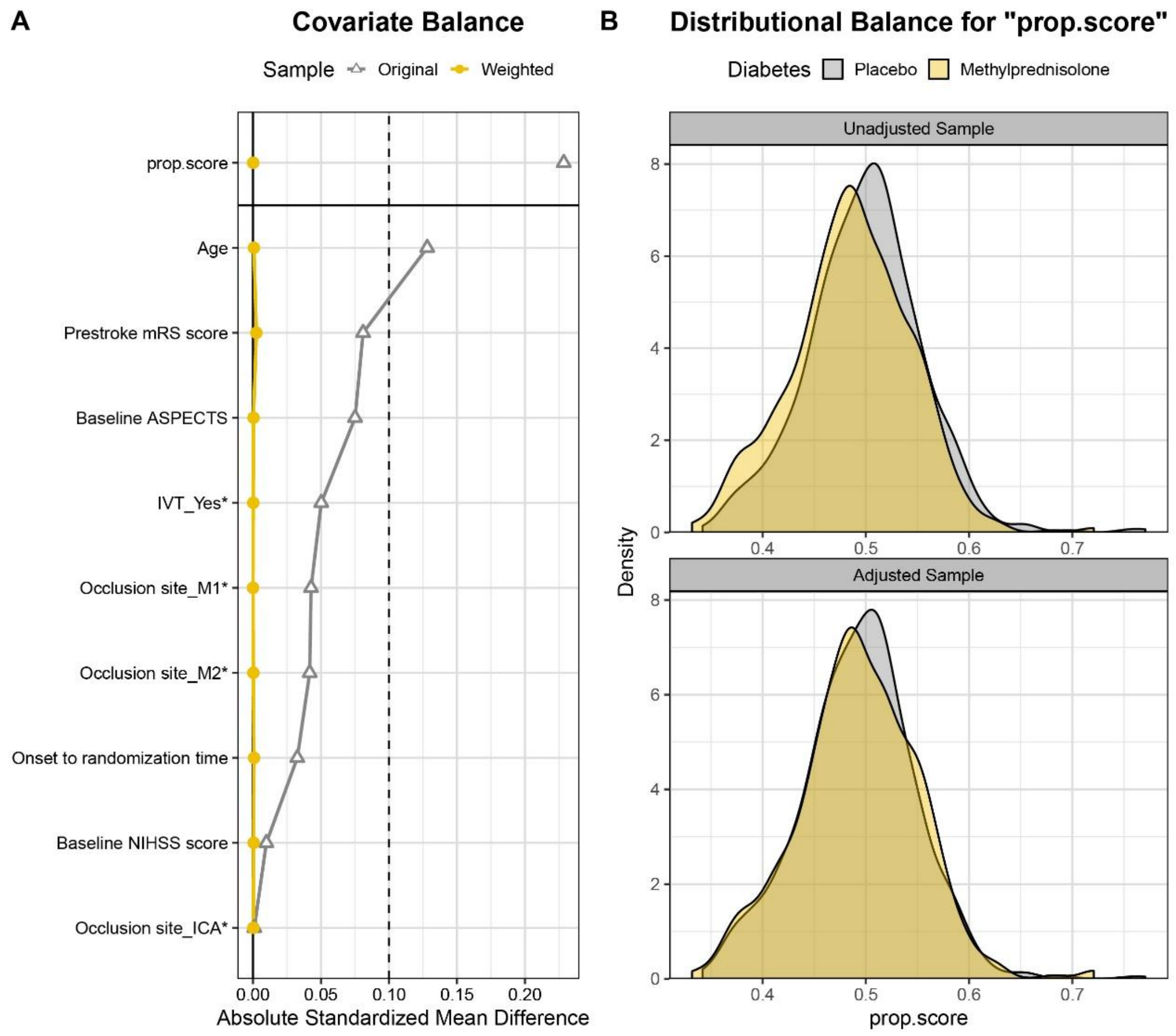

(A) Standardized mean differences before inverse probability of treatment weighting (gray) and after inverse probability of treatment weighting (yellow) for all selected baseline covariates. (B) Distributions of propensity scores before and after inverse probability of treatment weighting for the Methylprednisolone and control group.

Abbreviations: ASPECTS, Alberta Stroke Program Early Computed Tomography Score; EVT, endovascular treatment; ICA, internal carotid artery; IVT, intravenous thrombolysis; mRS, modified Rankin Scale; NIHSS, National Institutes of Health Stroke Scale.

Supplementary Figure 4. Inverse probability of treatment weighting for patients with admission hyperglycemia.

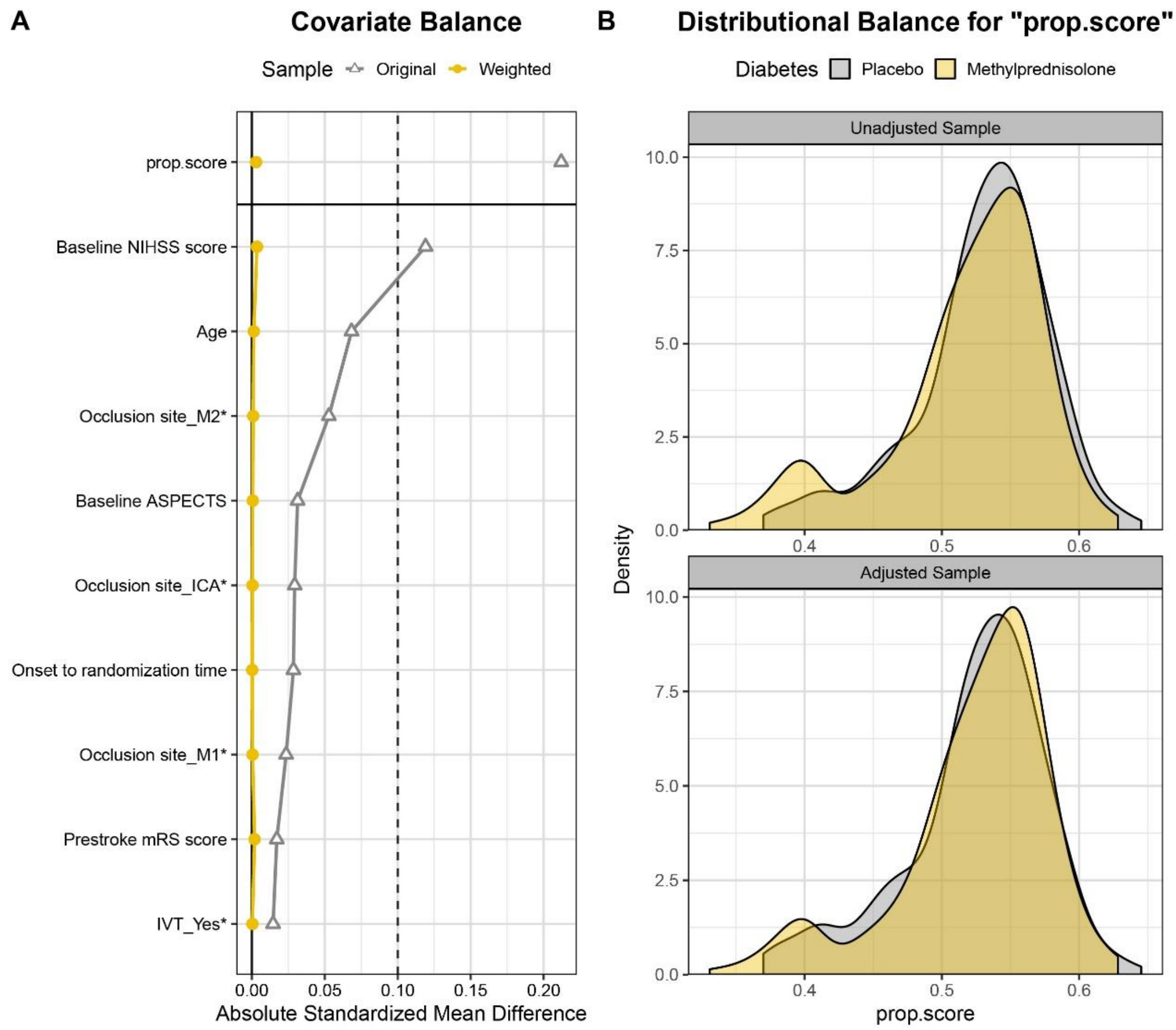

(A) Standardized mean differences before inverse probability of treatment weighting (gray) and after inverse probability of treatment weighting (yellow) for all selected baseline covariates. (B) Distributions of propensity scores before and after inverse probability of treatment weighting for the Methylprednisolone and control group.

Abbreviations: ASPECTS, Alberta Stroke Program Early Computed Tomography Score; EVT, endovascular treatment; ICA, internal carotid artery; IVT, intravenous thrombolysis; mRS, modified Rankin Scale; NIHSS, National Institutes of Health Stroke Scale.

Supplementary Figure 5. Inverse probability of treatment weighting for patients with admission hyperglycemia.

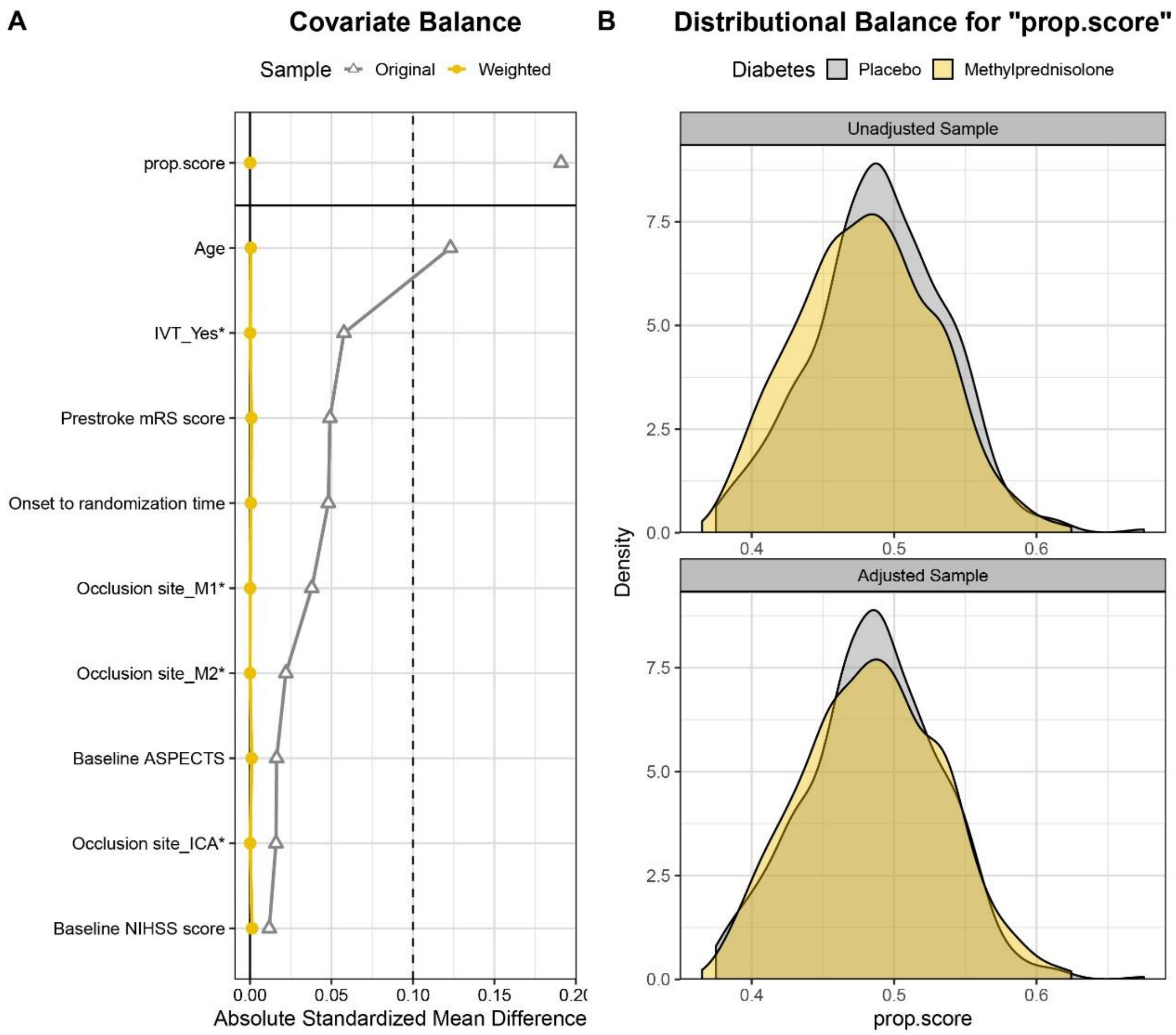

(A) Standardized mean differences before inverse probability of treatment weighting (gray) and after inverse probability of treatment weighting (yellow) for all selected baseline covariates. (B) Distributions of propensity scores before and after inverse probability of treatment weighting for the Methylprednisolone and control group.

Abbreviations: ASPECTS, Alberta Stroke Program Early Computed Tomography Score; EVT, endovascular treatment; ICA, internal carotid artery; IVT, intravenous thrombolysis; mRS, modified Rankin Scale; NIHSS, National Institutes of Health Stroke Scale.

Supplement Figure 6. Subgroup analysis of efficacy between methylprednisolone and placebo groups in patients with admission hyperglycemia.

Subgroup	Admission hyperglycemia patients		Adjusted OR (95% CI)	P for interaction
	Placebo	Methylprednisolone		
Overall	257	282	1.18 (0.87 - 1.61)	
Age				0.815
>69	137	150	1.24 (0.80 - 1.91)	
≤69	120	132	1.19 (0.76 - 1.86)	
Sex				0.451
Feman	133	141	1.05 (0.68 - 1.63)	
Men	124	141	1.33 (0.85 - 2.08)	
Baseline ASPECTS				0.707
>5	126	130	1.08 (0.69 - 1.68)	
≤5	129	150	1.25 (0.81 - 1.94)	
Baseline NIHSS score				0.280
>19	131	131	1.32 (0.83 - 2.09)	
≤19	126	151	1.03 (0.67 - 1.57)	
Intravenous thrombolysis				0.794
No	157	177	1.12 (0.76 - 1.67)	
Yes	100	105	1.31 (0.79 - 2.16)	
Time from onset to randomization				0.727
>362	123	130	1.22 (0.77 - 1.93)	
≤362	134	152	1.15 (0.75 - 1.76)	

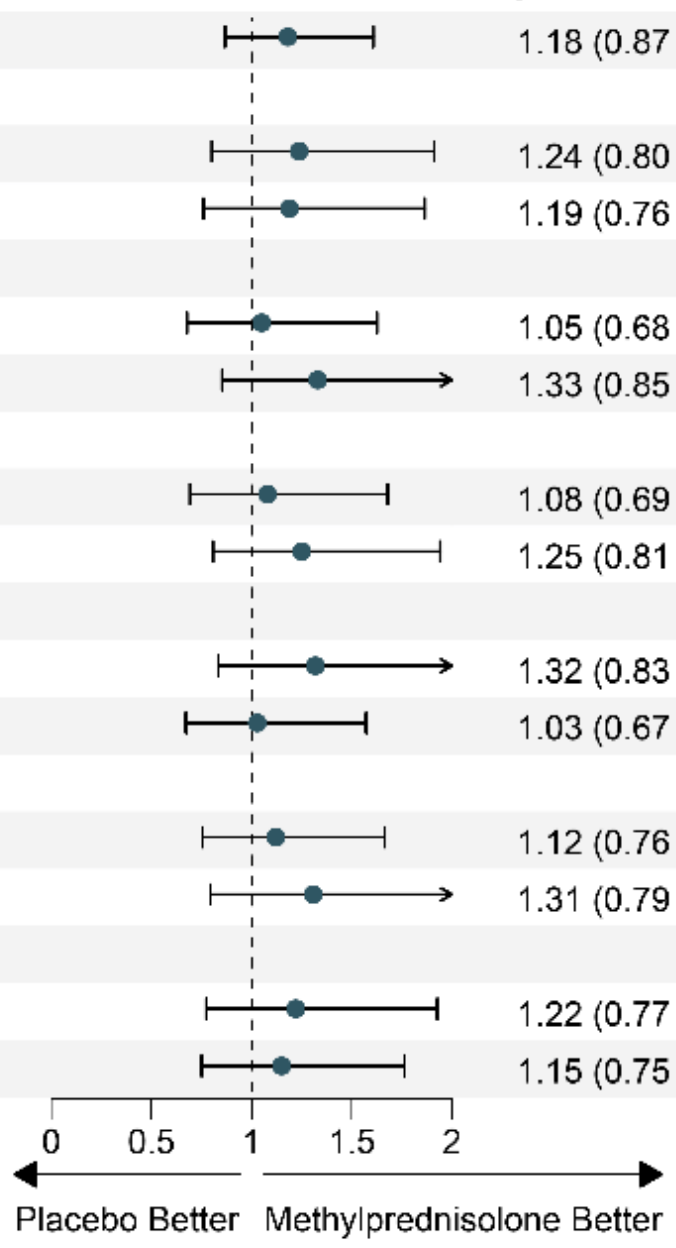

The forest plot shows adjusted odds ratios for the ordinal modified Rankin Scale score at 90 days across predefined subgroups. Models were adjusted for age, baseline NIHSS score, prestroke mRS score, baseline ASPECTS, intravenous thrombolysis, time from stroke onset to randomization, and occlusion location.

Abbreviations: ASPECTS, Alberta Stroke Program Early CT Score; CI, confidence interval; ICA, intracranial internal carotid artery; mRS, modified Rankin Scale; NIHSS, National Institutes of Health Stroke Scale; OR, odds ratio.

Supplement Figure 7. Subgroup analysis of efficacy between methylprednisolone and placebo groups in patients without diabetes.

	No diabetes patients			
**Subgroup**	**Placebo**	**Methylprednisolone**	**Adjusted OR (95% CI)**	**P for interaction**
Overall	686	665	1.20 (0.99 - 1.46)	
Age				0.854
>69	350	306	1.26 (0.95 - 1.67)	
≤69	336	359	1.15 (0.88 - 1.51)	
Sex				0.607
Feman	299	275	1.14 (0.85 - 1.54)	
Men	387	390	1.29 (1.00 - 1.67)	
Baseline ASPECTS				0.591
>5	355	324	1.12 (0.85 - 1.47)	
≤5	328	338	1.30 (0.98 - 1.71)	
Baseline NIHSS score				0.945
>19	312	296	1.25 (0.93 - 1.68)	
≤19	374	369	1.17 (0.91 - 1.51)	
Occlusion location				0.496
ICA	241	231	1.40 (1.01 - 1.96)	
M1	342	362	1.10 (0.85 - 1.44)	
M2	103	72	1.18 (0.68 - 2.06)	
Intravenous thrombolysis				0.143
No	408	430	1.08 (0.85 - 1.38)	
Yes	278	235	1.42 (1.03 - 1.95)	
Time from onset to randomization				0.270
>362	341	326	1.10 (0.83 - 1.44)	
≤362	345	339	1.27 (0.97 - 1.68)	

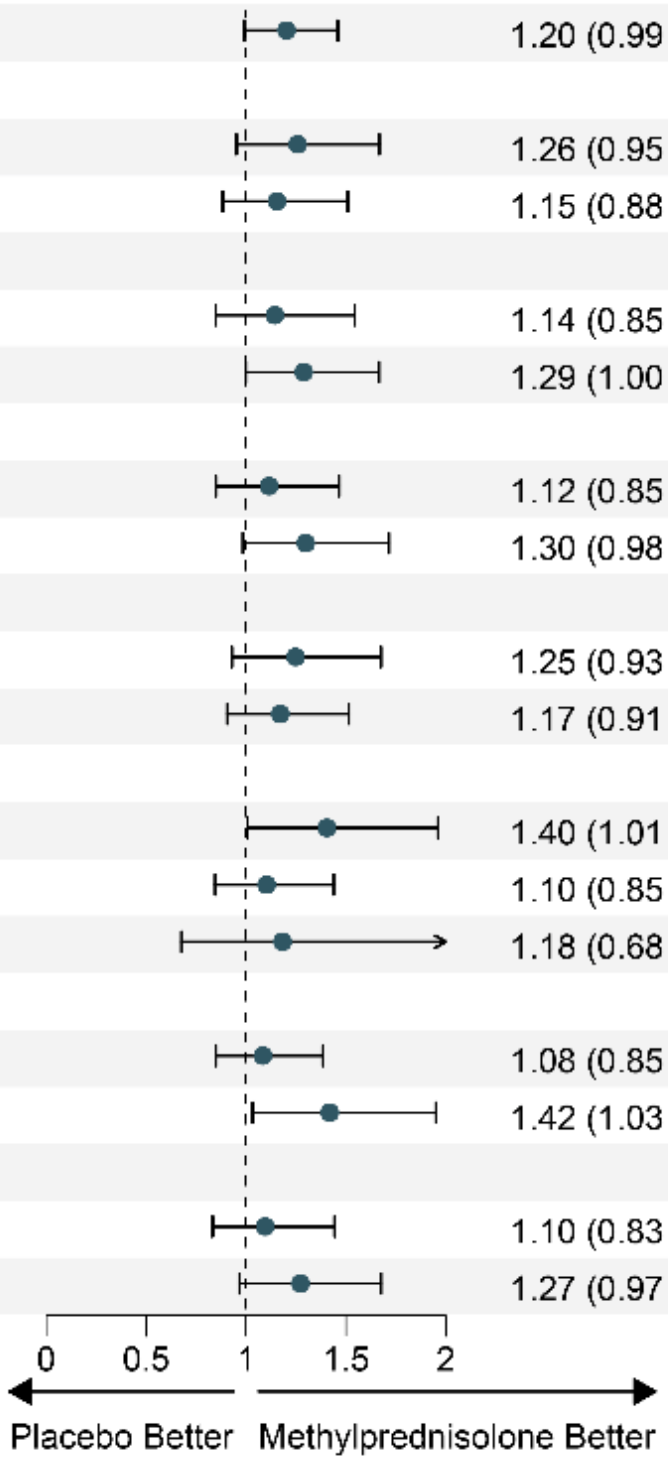

The forest plot shows adjusted odds ratios for the ordinal modified Rankin Scale score at 90 days across predefined subgroups. Models were adjusted for age, baseline NIHSS score, prestroke mRS score, baseline ASPECTS, intravenous thrombolysis, time from stroke onset to randomization, and occlusion location.

Abbreviations: ASPECTS, Alberta Stroke Program Early CT Score; CI, confidence interval; ICA, intracranial internal carotid artery; mRS, modified Rankin Scale; NIHSS, National Institutes of Health Stroke Scale; OR, odds ratio.

Supplement Figure 8. Subgroup analysis of efficacy between methylprednisolone and placebo groups in patients without admission hyperglycemia.

Subgroup	No Admission hyperglycemia patients: Placebo	Methylprednisolone	Adjusted OR (95% CI)	P for interaction
Overall	476	449	1.15 (0.91 - 1.45)	
Age				0.449
>69	227	189	1.12 (0.79 - 1.59)	
≤69	249	260	1.18 (0.86 - 1.61)	
Sex				0.504
Feman	182	175	1.10 (0.75 - 1.61)	
Men	294	274	1.26 (0.94 - 1.70)	
Baseline ASPECTS				0.416
>5	252	236	1.15 (0.84 - 1.59)	
≤5	223	212	1.16 (0.83 - 1.63)	
Baseline NIHSS score				0.540
>19	200	194	1.12 (0.78 - 1.60)	
≤19	276	255	1.17 (0.86 - 1.59)	
Occlusion location				0.917
ICA	168	149	1.22 (0.82 - 1.83)	
M1	238	244	1.12 (0.81 - 1.54)	
M2	70	56	1.16 (0.62 - 2.20)	
Stroke etiology				0.626
Large artery atherosclerosis	187	154	1.11 (0.75 - 1.63)	
Cardioembolism	224	228	1.26 (0.90 - 1.76)	
Others	22	17	3.44 (0.89 - 13.98)	
Unknown	43	50	1.16 (0.53 - 2.57)	
Intravenous thrombolysis				0.366
No	288	298	1.06 (0.79 - 1.42)	
Yes	188	151	1.31 (0.89 - 1.93)	
Time from onset to randomization				0.175
>362	250	234	1.00 (0.73 - 1.39)	
≤362	226	215	1.28 (0.91 - 1.79)	

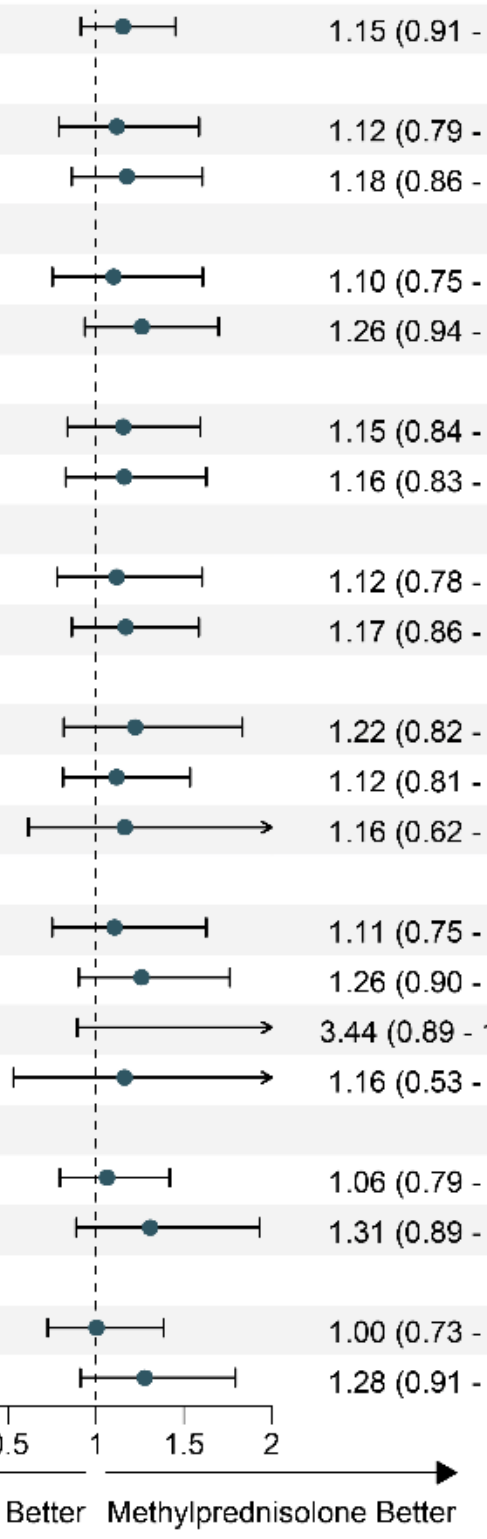

The forest plot shows adjusted odds ratios for the ordinal modified Rankin Scale score at 90 days across predefined subgroups. Models were adjusted for age, baseline NIHSS score, prestroke mRS score, baseline ASPECTS, intravenous thrombolysis, time from stroke onset to randomization, and occlusion location.

Abbreviations: ASPECTS, Alberta Stroke Program Early CT Score; CI, confidence interval; ICA, intracranial internal carotid artery; mRS, modified Rankin Scale; NIHSS, National Institutes of Health Stroke Scale; OR, odds ratio.

Supplement Figure 9. Margin effect.

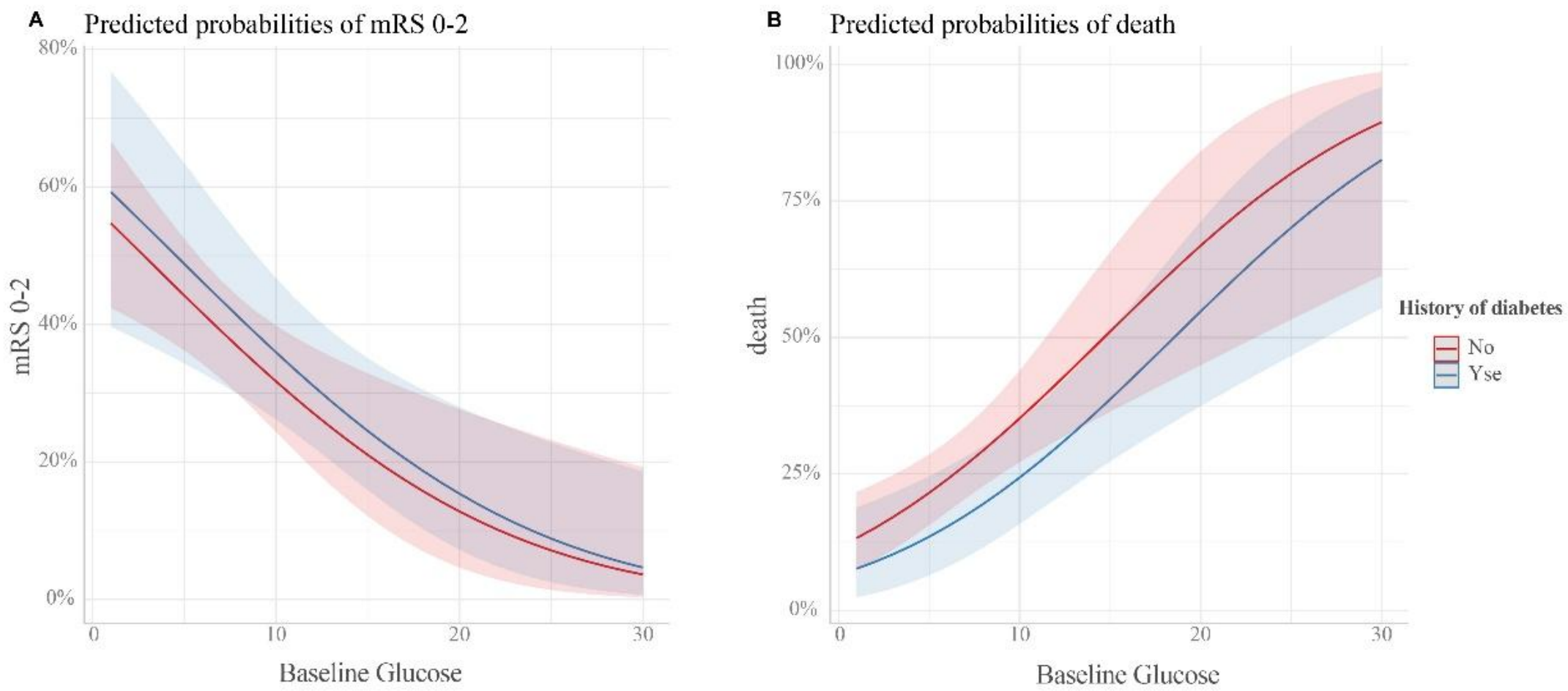

The association between baseline glucose and clinical outcomes (an mRS score of 0-2 and mortality) among patients stratified by the history of diabetes. The results demonstrated the predicted probability of an mRS score of 0-2 progressively decreased with increasing baseline glucose, while the predicted probability of mortality progressively increased in patients with or without diabetes mellitus.
